# The DEAD-box RNA-binding protein DDX6 regulates parental RNA decay for cellular reprogramming to pluripotency

**DOI:** 10.1371/journal.pone.0203708

**Published:** 2018-10-01

**Authors:** Daisuke Kami, Tomoya Kitani, Akihiro Nakamura, Naoki Wakui, Rena Mizutani, Masahito Ohue, Fuyuki Kametani, Nobuyoshi Akimitsu, Satoshi Gojo

**Affiliations:** 1 Department of Regenerative Medicine, Graduate School of Medical Science, Kyoto Prefectural University of Medicine, Kyoto, Japan; 2 Department of Cardiovascular Medicine, Graduate School of Medical Science, Kyoto Prefectural University of Medicine, Kyoto, Japan; 3 Department of Pediatric Cardiology and Nephrology, Graduate School of Medical Science, Kyoto Prefectural University of Medicine, Kyoto, Japan; 4 Department of Computer Science, School of Computing, Tokyo Institute of Technology, Tokyo, Japan; 5 Radioisotope Center, The University of Tokyo, Tokyo, Japan; 6 Department of Dementia and Higher Brain Function, Tokyo Metropolitan Institute of Medical Science, Tokyo, Japan; Korea University, REPUBLIC OF KOREA

## Abstract

Cellular transitions and differentiation processes require mRNAs supporting the new phenotype but also the clearance of existing mRNAs for the parental phenotype. Cellular reprogramming from fibroblasts to induced pluripotent stem cells (iPSCs) occurs at the early stage of mesenchymal epithelial transition (MET) and involves drastic morphological changes. We examined the molecular mechanism for MET, focusing on RNA metabolism. DDX6, an RNA helicase, was indispensable for iPSC formation, in addition to RO60 and *RNY1*, a non-coding RNA, which form complexes involved in intracellular nucleotide sensing. RO60/*RNY1*/DDX6 complexes formed prior to processing body formation, which is central to RNA metabolism. The abrogation of DDX6 expression inhibited iPSC generation, which was mediated by RNA decay targeting parental mRNAs supporting mesenchymal phenotypes, along with microRNAs, such as miR-302b-3p. These results show that parental mRNA clearance is a prerequisite for cellular reprogramming and that DDX6 plays a central role in this process.

## Introduction

The Ro 60-kDa protein (RO60), which is a ring-shaped RNA-binding protein consisting of α-helical HEAT repeats, was initially identified as a target of the immune response in patients with systemic lupus erythematosus [1]. *RNY*s (Y RNAs), most of which are assembled into Ro ribonucleoproteins (RNPs), were first characterized in humans as ~100-nucleotide noncoding RNAs [2]. Ro possesses two distinct RNA-binding sites, one binds to Y RNA with high affinity and non-sequence-specific RNAs with low affinity on the outer surface, and the other is the central tunnel for 3′ single-stranded extension [3]. The binding site for Y RNA also overlaps with a nuclear accumulation signal; therefore, when Y RNA binds to RO60, it occludes the nuclear accumulation signal, resulting in the retention of the RO60-bound to Y RNA in the cytoplasm [4]. A model for RNA decay via a Ro ortholog (RO60 related: Rsr), Y RNA, and exoribonuclease polynucleotide phosphorylase (PNPase) has been proposed. The association of PNPase with Rsr via Y RNA, to form RNA degradation machinery, called RYPER, changes the conformation of Y RNA on Rsr as a competitor for other RNAs, rendering the cavity of Rsr accessible to RNA substrates [5]. In mammals, PNPase exists in the mitochondrial intermembrane space, and RYPER cannot form in the cytoplasm; RNA decay by RYPER might involve other mechanisms [6].

The translation and decay of mRNAs play essential roles in gene expression regulation, not only for the maintenance of homeostasis but also to ensure survival in changing environments; dysregulation might result in cell death. General mRNA decay pathways are initiated by the shortening of the 3′ polyA tail of the Ccr4/Pop/Not complex, which is catalyzed by deadenylase [7], followed by either 3′-to-5′ degradation by exosomes [8] or decapping by Dcp1/Dcp2 to promote 5′-to-3′ exonucleolytic decay [9]. Small non-coding RNA (ncRNA) molecules of approximately 20–22 nucleotides, termed miRNAs, regulate gene expression via RNA-induced silencing complexes (RISCs) [10]. Regulation involves either mRNA decay or translational repression [11]. The 3′-terminal mRNA decay complex, including GW182, Ago, and two deadenylase complexes, CCR4-NOT and PAN2-PAN3 [12], not only carry out polyA shortening but also promote the dissociation of polyA-binding protein (PABP) from target mRNA to increase the accessibility of the polyA tail to deadenylases [13], and they are bridged to 5′ m^7^G-cap via DDX6, a decapping activator [14]. This results in 5′ to 3′ exonucleolytic decay by XRN1. These molecules, including miRNAs, XRN1, DCP2, AGO, GW182, and DDX6, localize to mRNA-processing bodies (P-bodies), which are dynamic components in the cytoplasm, depending on the pool of non-translating mRNAs [15] and RNA decay intermediates [16].

Pluripotent stem cells have the unique abilities to self-renew and differentiate into diverse cell types. Four key transcription factors, OCT4, SRY-box2 (SOX2), Krüppel-like factor 4 (KLF4), and MYC (collectively known as OSKM), determine differentiation potential and are involved in the generation of induced pluripotent stem cells (iPSCs) [17]. The reprogramming process to iPSCs includes three distinct phases, i.e., initiation, maturation, and stabilization [18]. The initial phase involves the mesenchymal-to-epithelial transition (MET), which is mediated by the suppression of Snail and activation of E-cadherin [19]. In addition, proteins related to mRNA decay are induced during this initiation phase, and reprogramming induction by OSKM inhibits genes specifying the differentiated identity [20].

We characterized a novel obstacle for cellular reprogramming based on ncRNA expression and identified a previously unreported role for the RO60/*RNY1*/DDX6 complex. Since each constituent of the complex was involved in RNA decay, which is critical for various biological processes, we determined whether and how the complex functions to generate iPSCs.

## Results

### RNY1 plays a regulatory role in the cellular reprogramming process

**Fig 1:**
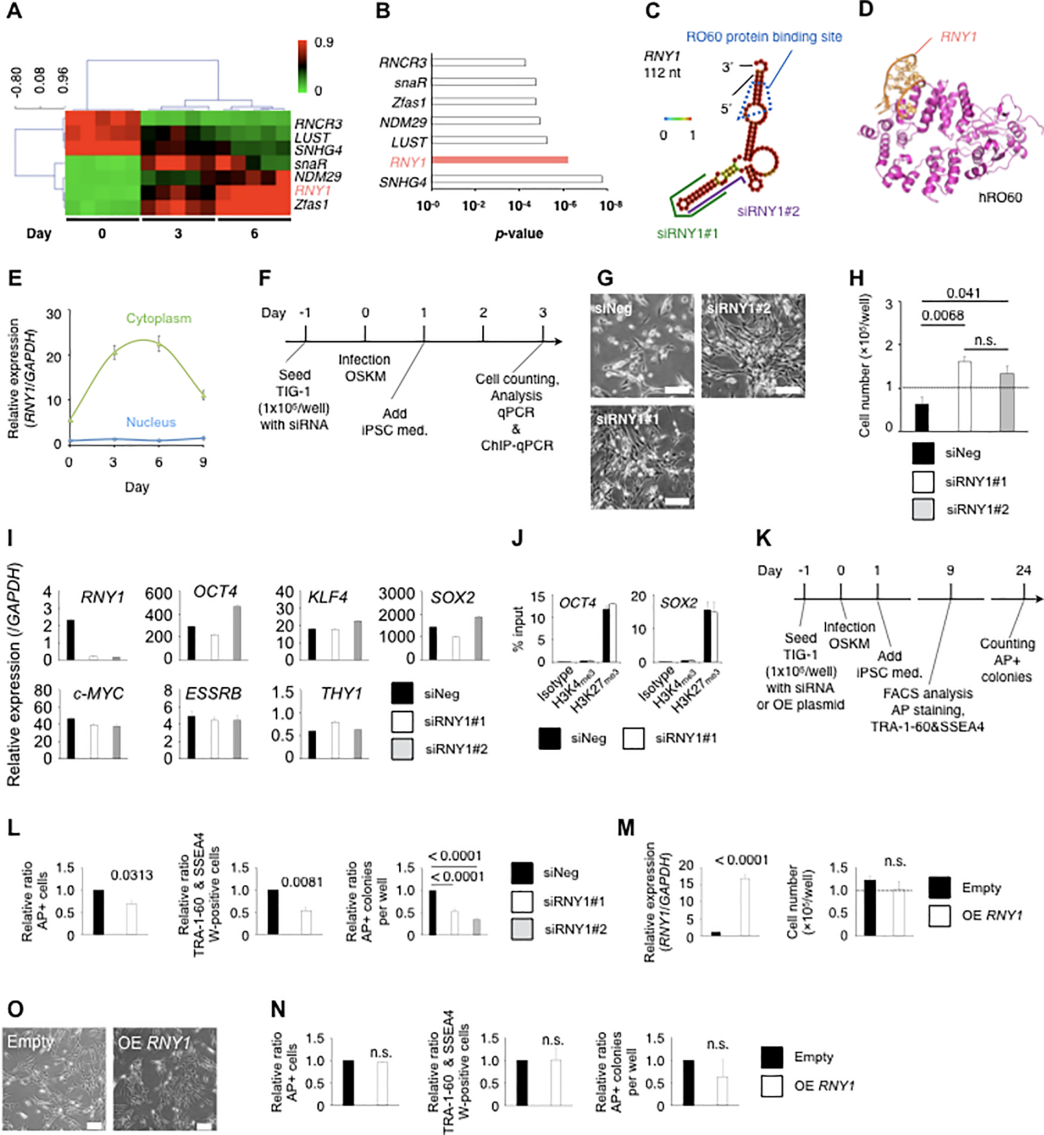
Analysis of non-coding RNAs during the early stage of iPS reprogramming. (A) Heat map showing non-coding RNA expression at various time points during iPS reprogramming obtained using the LncProfiler qPCR Array Kit. Graph showing *RNY1* gene expression during iPS reprogramming. Statistical differences were assessed by t-test and standard Bonferroni correction between Day 0 and Days 3 and 6. (B) Statistical analysis of non-coding RNA expression during iPS reprogramming. (C) Secondary structure of *RNY1* from computational prediction. (D) Structure of human RO60 from computational modeling. (E) RNA expression of *RNY1* in the cytoplasm and nucleus of OSKM-treated TIG-1 fibroblasts. Individual RNA expression levels were normalized to *GAPDH* expression levels. Data are presented as the mean ± SEM. (F) Schematic representation of the early iPS reprogramming analysis using the transient knockdown method on Day 3 for (G) to (J). Med: medium. OSKM: OCT4, SOX2, KLF4, and c-MYC. (G) Phase contrast micrograph images of siRNA for Negative control, RNY1#1-, RNY1#2-, and OSKM-treated TIG-1 fibroblasts after 3 days. The white bar indicates 200 μm. (H) Cell numbers for OSKM- and siRNA-treated TIG-1 fibroblasts on Day 3. (I) RNA expression in OSKM- and siRNA-treated TIG-1 fibroblasts on Day 3. Individual RNA expression levels were normalized to *GAPDH* expression levels. Data are presented as the mean ± SEM. (J) ChIP-qPCR analysis of iPS reprogramming on Day 3. (K) Schematic representation of the iPSC reprogramming analysis using the transient knockdown method on Days 9 and 24. AP: alkaline phosphatase. (L) Efficacy of iPS reprogramming with siRNAs by flow cytometry using SSEA4 and TRA-1-60 antibodies, AP staining on Day 9, and AP-positive colony counting of iPSCs on Day 24. (M) Cell numbers and *RNY1* expression levels of *RNY1*-overexpressing TIG-1 fibroblasts 3 days after transfection. OE: Overexpression. (N) Efficacy of iPSC reprogramming with an overexpression plasmid by flow cytometry using SSEA4 and TRA-1-60 antibodies, AP staining on Day 9, and colony counting of iPSC colonies using AP staining on Day 24. OE: overexpression. (O) Phase contrast micrograph images of *RNY1*-overexpressing TIG-1 fibroblasts after 3 days. The white bar indicates 200 μm.

To elucidate the regulatory mechanism for cellular reprogramming at the early stage, we examined ncRNA expression in a human fetal fibroblast cell line, TIG-1 [21]. A time-course analysis of ncRNA expression in OSKM-transduced TIG-1 fibroblasts was performed (Fig 1A, S1 Table). We identified 49 ncRNAs expressed at the early stage (i.e., before Day 6) (S1 Table). Among them, *RNY1*, which is a small ncRNA (112 nucleotides), was strongly expressed only in the early stage, exhibiting the most significant difference over time (Fig 1B, S2 Table). *RNY1* was initially identified as an RNA component of RNPs, and it is associated with the autoimmune antigen proteins RO60 and SSB [22]. *RNY1* is highly conserved in vertebrates to bacteria, and the RO60-binding sequences are particularly well conserved [23]. In the present study, a computational structure analysis indicated that *RNY1* contains loop structures and a double-stranded stem region formed by pairing of the 5′ and 3′ ends. Eighty-eight percent of nucleotides were paired, indicating a robust secondary structure (Fig 1C). This stem contains a bulged helix region critical for binding to RO60 during RNP formation [24]. Computational structure modeling revealed that human RO60, which has 78% protein sequence identity with that of *Xenopus*, had a nearly identical 3D structure, including the *RNY1*-binding region [25] (Fig 1D). Previous reports have suggested that interactions of RO60 with *RNY1* protect and stabilize *RNY1* [26]; however, the binding of *RNY1* to RO60 conceals the nuclear accumulation signal of RO60, resulting in the transport of the complex to the cytoplasm [4]. Two short-interfering RNAs (siRNAs) targeting *RNY1* designed to knock down its expression were mapped in this secondary structure (Fig 1C). *RNY1* in OSKM-transduced fibroblasts was sharply increased in the cytoplasm fraction at the early stage of iPS reprogramming and peaked between Days 3 and 6, based on quantitative reverse transcriptase polymerase chain reaction (qPCR) (Fig 1E). In contrast, *RNY1* in the nuclear fraction exhibited a minimal increase. Thus, under these reprogrammed conditions, *RNY1* might function in the cytoplasm.

We examined the role of *RNY1* in the early stage of reprogramming using loss- or gain-of-function experiments over the time line shown in Fig 1F (Fig 1G–1J). *RNY1-*knockdown fibroblasts were markedly different with respect to cell shape (Fig 1G) and significantly different with respect to cell number on Day 3 (Fig 1H) compared with fibroblasts transfected with scramble siRNA. *RNY1-*knockdown fibroblasts maintained the parental morphological characteristics and were resistant to apoptosis, which is induced in the early reprogramming process. We determined whether *RNY1-*knockdown affects key transcriptional networks for reprogramming using two siRNAs targeting *RNY1* and siRNAs with scrambled sequences in OSKM-transduced fibroblasts to exclude non-specific effects, such as proliferation and transcriptome effects. Both siRNAs knocked down *RNY1* expression to approximately 10% of the levels in the control. Pluripotent stem cell markers, e.g., *OCT4*, *KLF4*, *SOX2*, and *ESSRB*, did not differ between the two *RNY1-*knockdown fibroblasts and the negative control on Day 3 (Fig 1I). To determine whether *RNY1* is involved in chromatin modification, we used chromatin immunoprecipitation-qPCR (ChIP-qPCR) targeting the *OCT4* and *SOX2* promoter regions for both H3K4me3 and H3K27me3. There were no significant differences between *RNY1-*knockdown fibroblasts and the negative control for both chromatin modifications in the promoter regions of both transcription factors on Day 3 (Fig 1J).

The efficacy of iPS reprogramming in siRNA1#1-treated fibroblasts was significantly decreased, based on TRA-1-60 and SSEA4 double-positive cells or alkaline phosphatase (AP)-positive cells on Day 9, and the efficacy of iPS reprogramming in either siRNY1#1- or #2-treated fibroblasts was significantly decreased, based on the numbers of AP-positive colonies on Day 24 (Fig 1K and 1L). Following the over-expression of *RNY1* during iPS reprogramming, there were no differences in cell shape, cell growth, and iPS formation efficacy compared with those in mock transfectants (Fig 1M and 1O). Accordingly, *RNY1* might play a crucial in cellular reprogramming, but its effect may reach saturation.

### RO60 and DDX6 form protein complexes

**Fig 2:**
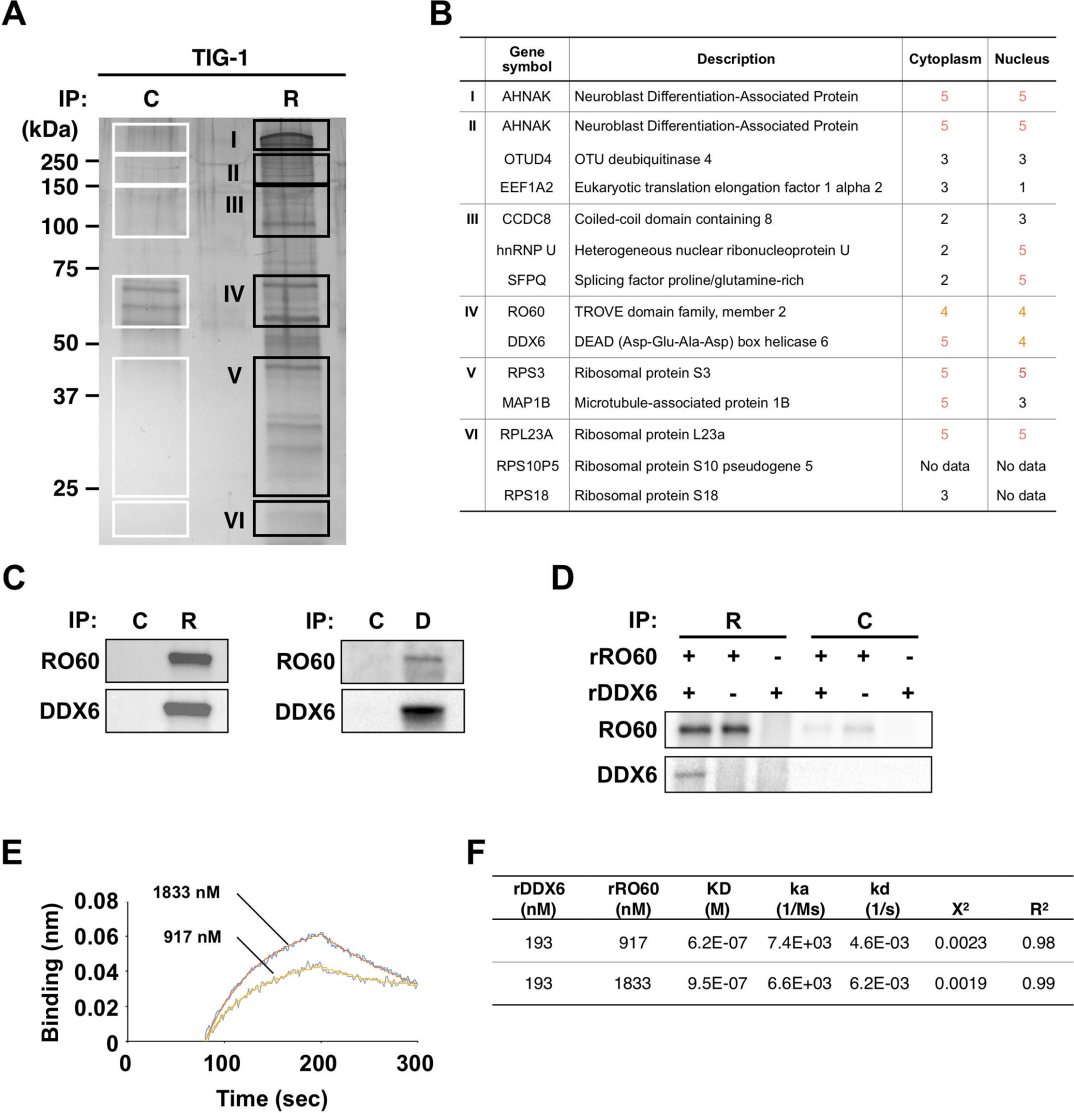
Identification of the functions of RO60 and DDX6 during iPS reprogramming. (A) Silver staining of immunoprecipitated proteins using the RO60 antibody. C: Control antibody; R: RO60 antibody. (B) Candidate proteins that interact with RO60. Protein localization was evaluated using GeneCards (http://www.genecards.org). (C) Immunoprecipitation (IP) of protein extracts from OSKM-transduced TIG-1 fibroblasts with magnetic beads coupled to RO60 or DDX6 antibodies or to an irrelevant isotype-matched control antibody, followed by an immunoblot analysis with RO60 or DDX6 antibodies. Graphs showing *RNY1* abundances in immunoprecipitated TIG-1 proteins determined by qPCR. C: Control antibody. R: RO60 antibody. D: DDX6 antibody. (D) Immunoblotting analysis of rDDX6 and rRO60 binding assays *in vitro* with RO60 and DDX6 antibodies. (E) Binding assays of rDDX6 to rRO60 using the BLItz system. Whole rDDX6 binding to rRO60. Binding kinetics of rDDX6 was titratable to 193 nM. (F) Binding assay results for rDDX6 to rRO60.

To investigate the connection between *RNY1*-RO60 and the factors involved in iPS remodeling, we identified the RO60-associating factors using immunoprecipitation (IP), followed by mass spectrometry (MS) analysis. Using IP and MS (Fig 2A), we identified 12 candidate cytoplasmic proteins that interact with RO60 in the stationary phase of TIG-1 fibroblasts (Fig 2B). Among these, DDX6, Neuroblast Differentiation-Associated Protein (AHNAK), and Microtubule-associated protein 1B (MAP1B) were present in the cytoplasm where the *RNY1*-RO60 complex is localized, but they are not ribosomal proteins, according to Human Gene Database GeneCards^®^ (http://www.genecards.org). The protein encoded by AHNAK was a large scaffold of 700 kDa, and MAP1B was 270 kDa. Neither immunoprecipitated candidate was observed in the expected molecular weight region. Only DDX6 was detected in the expected molecular weight region, indicating an interaction between *RNY1*-RO60 and DDX6 during early reprogramming.

First, we evaluated the interaction between RO60 and DDX6 in fibroblasts using IP at the growth phase, not in the reprogramming setting. DDX6 protein complexes were strongly co-immunoprecipitated by an anti-RO60 antibody, and RO60 protein complexes were also co-immunoprecipitated by an anti-DDX6 antibody (Fig 2C). We estimated the endogenous amounts of RO60 and DDX6 to be 3.21 and 456 fmol/μg protein, respectively, by comparing the western blotting signals between recombinant purified recombinant proteins and endogenous proteins (Part A of S1 Fig). The molecular ratio of RO60 to DDX6 was approximately 1:142 in the whole cell component and approximately 1:120 in the cytoplasmic component (Part B of S1 Fig).

We analyzed the functional avidity of the RO60 and DDX6 proteins using an *in vitro* binding assay with recombinant proteins, rRO60 and rDDX6. RO60-IP for various combinations of rDDX6 and rRO60 revealed *in vitro* complex formation between rDDX6 and rRO60 (Fig 2D). We assessed binding using a biolayer interferometry method with the BLItz system [27]. The *K*_D_ values for 193 nM rDDX6 and 917 nM or 1833 nM rRO60 were 6.2 × 10^−7^ M and 9.5 × 10^−7^ M, respectively (Fig 2E and 2F). These data indicated that rRO60 bound to rDDX6 in a concentration-dependent manner, suggesting that these proteins were specific binding partners. Our results clearly demonstrated that DDX6 and RO60 can assemble both *in vivo* and *in vitro*. Both proteins are involved in RNA metabolism, and these results might also link them to a single axis that coordinately functions in RNA metabolism depending on the assembly state.

### DDX6 is released from RO60/*RNY1* complexes during the early reprogramming stage and forms P-bodies involved in RNA metabolism

**Fig 3:**
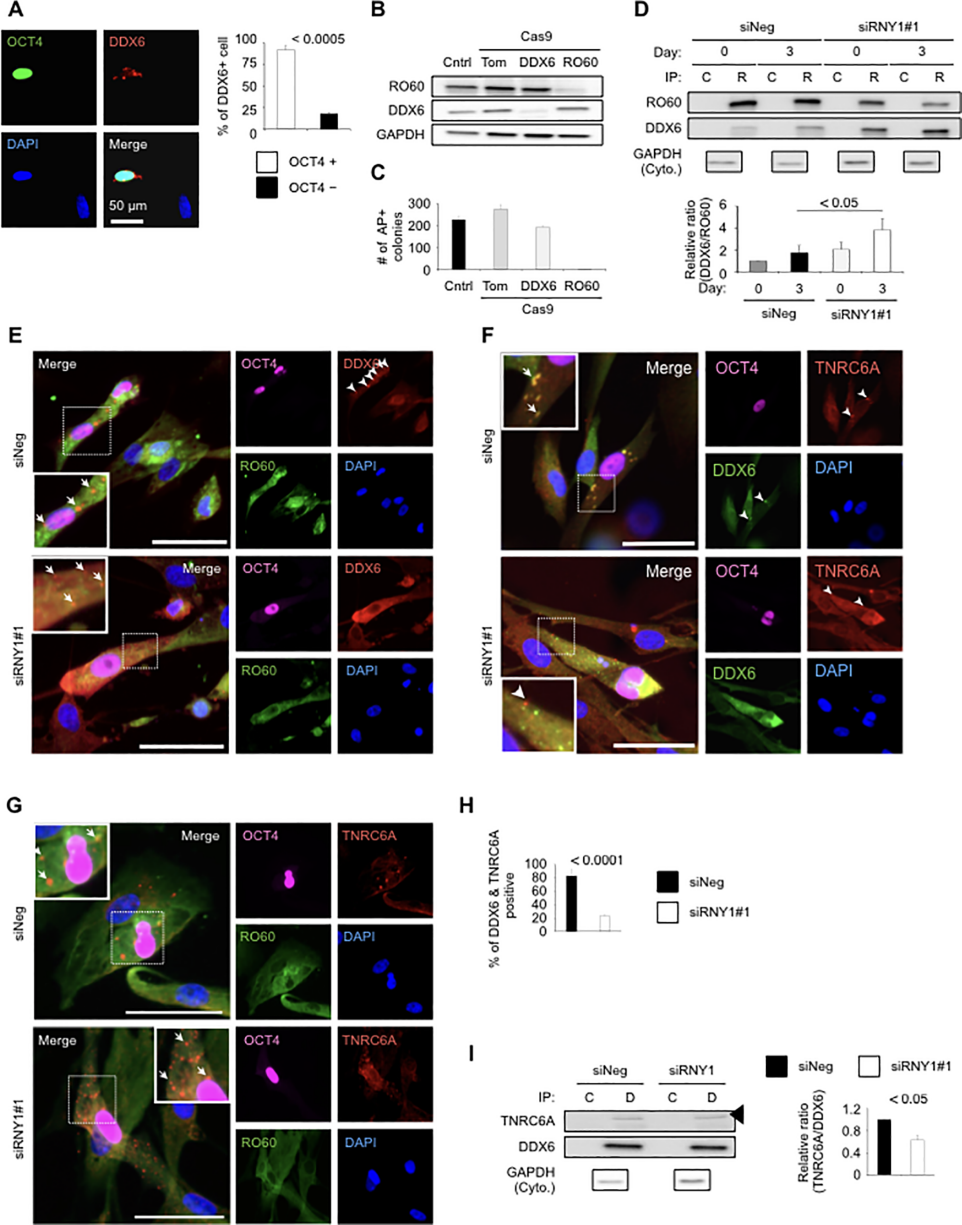
DDX6-RO60 complexes were regulated by *RNY1*. (A) Immunocytochemistry of OSKM-treated TIG-1 fibroblasts on Day 3. Graph showing the percentage of DDX6-positive cells. (B) Immunoblotting analysis of Cas9-treated TIG-1 fibroblasts. Tom: tdTomato as a mock control. (C) iPS reprogramming of Cas9-treated TIG-1 fibroblasts. AP-positive colonies were counted on Day 20. (D) Immunoblotting analysis of RO60-IP proteins from OSKM- and siRNA-treated TIG-1 fibroblasts with RO60, DDX6, and GAPDH antibodies. The raw blotting data are attached to S8 Fig. (E) Immunocytochemical analyses of RO60, DDX6, and OCT4 in OSKM- and siRNA-treated TIG-1 fibroblasts. (F) Immunocytochemical analyses of TNRC6A, DDX6, and OCT4 in OSKM- and siRNA-treated TIG-1 fibroblasts. (G) Immunocytochemical results for RO60, TNRC6A, and OCT4 in OSKM- and siRNA-treated TIG-1 fibroblasts. (H) Immunoblotting analysis of DDX6-IP proteins from OSKM- and siRNA-treated TIG-1 fibroblasts with TNRC6A, DDX6, and GAPDH antibodies. The white bar indicates 50 μm in (A), (E) to (G).

During the reprogramming process on Day 3, more than 86% of fibroblasts with OCT4 expression exhibited co-staining with an anti-DDX6 antibody, which accumulated to form specific spots with diameters of a few micrometers (Fig 3A). We next determined whether RO60 or DDX6 regulated the cellular reprogramming process using a lentivirus carrying clustered regularly interspaced short palindromic repeats (CRISPR)/Cas9 and single guide RNA for RO60, DDX6, and tdTomato (Tom) (Fig 3B). All transduced fibroblasts had longer population doubling times than that of the parental cells, but the population doubling times did not differ significantly among the three types of cells (Part C of S1 Fig). Cells formed embryonic stem-like colonies with counts of 226 ± 14.0, 274.2 ± 21.2, and 192.3 ± 6.1, on average, for the parental cells, mock-transfected cells, and RO60-knockout cells, respectively, 20 days after OSKM induction. DDX6-knockout cells formed few colonies (2.4 ± 0.8), and those that formed exhibited an abnormal collapsed morphology (Fig 3C and Part D of S1 Fig). RO60-knockout cells did not significantly affect cellular reprogramming, whereas DDX6 depletion completely abrogated the ability of fibroblasts to be reprogrammed to iPSCs, indicating that DDX6 is indispensable for this process.

To dissect the kinetics of DDX6 and RO60 during reprogramming after *RNY1-*knockdown, we performed qPCR, immunoblotting, and immunohistochemical analyses. There were no significant differences in *RO60* and *DDX6* expression levels between control and *RNY1-*knockdown fibroblasts following OSKM transfer or mock transfer (Part A of S2 Fig). Cytoplasmic RO60 expression was significantly decreased in *RNY1-*knockdown cells but still persisted at about 22.0 ± 0.8% (Part B of S2 Fig). Both siRNY1#1 and #2 were targeted to the distal stem of the secondary structure; the proximal stem of the secondary structure contains the binding sequence for the outer surface of the HEAT repeats of RO60 and conceals the nuclear accumulation signal (Fig 1C and 1D). As demonstrated previously [4], after siRNA-mediated knockdown, some *RNY1* might remain on RO60, resulting in the partial persistence of RO60 in the cytoplasm.

An RO60 immunoprecipitation analysis at the early reprogramming stage revealed that DDX6 is released from RO60 (Fig 3D), although RO60 was bound to DDX6 in the growth phase (Fig 2C). On the other hand, *RNY1-*knockdown cells exhibited greater binding of RO60 to DDX6 than that in the native growth phase. The persistence of the binding of DDX6 to RO60 might inhibit movement into P-bodies, whereas the dissociation of DDX6 from RO60 might enable the formation of P-bodies along with many proteins, including miRISCs.

We investigated the subcellular distribution of DDX6 and RO60 at the early reprogramming stage using immunocytochemistry. The subcellular localization of DDX6 is dynamic across the cytoplasm and the nucleus [28]. DDX6 is concentrated in P-bodies in HeLa cells [29]; we examined several cell lines (TIG-1 fibroblasts and 3T3-L1 preadipocytes) using immunofluorescence to clarify the diffuse cytoplasmic distribution (Part C of S2 Fig). DDX6 in OCT4-expressing control fibroblasts formed P-body structures, referred to as GW182 ortholog TNRC6A proteins, in the cytoplasm, and DDX6 in OCT4-expressing *RNY1-*knockdown fibroblasts, which was rare, exhibited diffuse staining with a few small aggregates (Fig 3E and 3F). RO60 was diffused in the cytoplasm in the control as well as *RNY1-*knockdown cells, which was consistent with a previous report [4], and did not co-localize with TNRC6A (Fig 3E–3G). To investigate the co-localization rate of DDX6 and TNRC6A during iPS reprogramming, we counted the co-localization rates. This result shows that the co-localization rate of DDX6 and TNRC6A on siRNY1-treated cells was significantly decreased (Fig 3H). Furthermore, to determine the influence of *RNY1* on TNRC6A, we examined DDX6-IP in *RNY1-*knockdown fibroblasts on Day 3. The amount of TNRC6A that was bound to DDX6 was significantly lower in *RNY1-*knockdown cells than that in the control cells transfected with siNeg (Fig 3I).

### Global gene expression analysis

We compared global gene expression in RNY1-knockdown and control TIG-1 fibroblasts on Day 3 during OSKM transduction using the Agilent Human Microarray Chip (S3 Fig, S3 Table). These results support the data by indicating higher rates of cell growth in RNY1-knockdown fibroblasts than in control fibroblasts. The gene cluster related to RNA metabolism decreased in RNY1-knockdown fibroblasts, supporting our hypothesis that RNY1 regulates mRNAs, especially the degradation or silencing of pre-existing mRNA pools (Part D of S3 Fig). Further details are described in the S3 Fig legend.

### DDX6-IP included iPS reprogramming-related miRNAs that regulate MET

**Fig 4:**
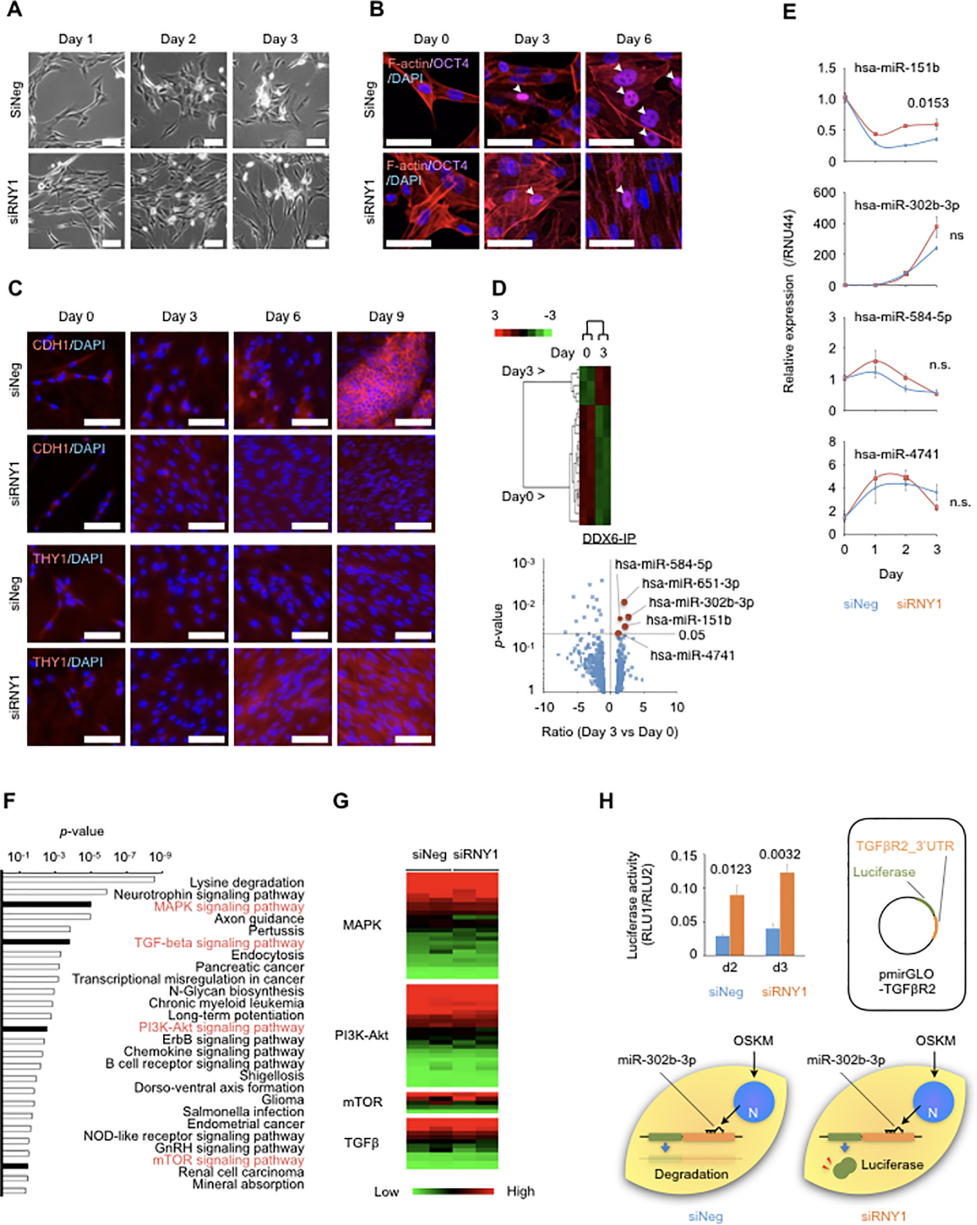
miRNA analysis of iPS reprogramming. (A) Phase contrast microscopy images of OSKM- and siRNA-treated TIG-1 fibroblasts from Days 1 to 3. Immunocytochemistry analysis on Day 3 using OCT4 antibody and CytoPainter Phalloidin-iFluor 594. The white bar indicates 200 μm. (B) Immunocytochemical analyses of OCT4, DAPI, and F-actin in OSKM- and siRNA-treated TIG-1 fibroblasts. Arrowheads indicate OCT4-positive cells. The white bar indicates 50 μm. (C) Immunocytochemical analyses of DAPI and CDH1 or THY1 in OSKM- and siRNA-treated TIG-1 fibroblasts. The white bar indicates 100 μm. (D) Heat map of miRNA expression of DDX6-IP in TIG-1 fibroblasts using the nCounter system. These miRNAs were significantly expressed. Volcano plot of global miRNA expression in DDX6-IP proteins in OSKM-treated TIG-1 fibroblasts using the nCounter system. (E) Mature miRNA expression in OSKM- and siRNA-treated TIG-1 fibroblasts from Days 1 to 3. Individual RNA expression levels were normalized to the respective *RNU44* expression levels. Data represent the mean ± SEM. (F) Target genes of miRNAs in DDX6-IP proteins were categorized based on KEGG pathways. Red letters indicate iPS reprogramming-related signaling pathways. (G) Heat map of mRNA expression in whole TIG-1 fibroblast lysates. (H) Luciferase activity using hsa-miR-302b-3p targeting the pmirGLO plasmid carrying the 3′ UTR of TGFβR2. d2 and d3 indicates 2 or 3 days after OSKM induction.

Control fibroblasts changed dramatically from spindle-shaped to epithelial-like forms at the early reprogramming stage (Fig 4A), indicating that the process of MET had occurred. In contrast, there were no morphological changes in *RNY1-*knockdown fibroblasts (Fig 4A). We observed drastic morphological differences between *RNY1-*knockdown and control fibroblasts using fluorescent staining with iFluor 594-labeled Phalloidin against F-actin (Fig 4B). The former type clearly exhibited the parental mesenchymal morphology, whereas the latter completely acquired the epithelial morphology only 3 days after OSKM transfer.

To analyze the proteins that increased during iPS reprograming, we performed immunocytochemistry analyses of CDH1, which is a marker of epithelial cells, and THY1, which is a marker of fibroblasts (Fig 4C). CDH1 proteins in control cells were clearly expressed after Day 6, whereas those in *RNY1-*knockdown cells were negligibly expressed. However, THY1 proteins in control cells were weakly expressed, whereas those in *RNY1*-knockdown cells were produced and increased over time.

DDX6 contributes to several aspects of RNA metabolism, such as 5′ decapping, bridging between 5′ decapping and 3′ deadenylation machinery, and miRNA-mediated RNA silencing [30]. During cellular reprogramming to iPSCs, miRNAs play an indispensable role. In addition, MET involves the clearance of mesenchymal transcripts, and this process is partially regulated by miRNAs. We analyzed miRNAs coupled with DDX6-IP proteins (Part A of S4 Fig, Fig 4D, and S4 Table) and whole cell proteins (Parts B and C of S4 Fig) in OSKM-treated fibroblasts on Day 3 using the nCounter system. We examined 5 miRNAs based on *p*-values. miR-302b-3p, expressed in iPSCs, exhibited the greatest change from Day 0 to Day 3. In addition, miRNAs from whole cytoplasmic samples included miR-302a-3p, miR-302b-3p, miR-302c-3p, miR-367-3p, and others (Parts B and C of S4 Fig), indicating that miRNA-mediated RNA silencing, involving DDX6, was associated with reprogramming. To analyze the changes in miRNA expression in detail over time, we examined OSKM- and siRNA-treated fibroblasts on Days 1, 2, and 3 using TaqMan PCR. There were no significant differences in miRNA levels between the cell types, except for has-miR-151b, which exhibited lower expression in treated cells than in control cells (Fig 4E). The pathway involving *RNY1* did not affect the expression levels of miRNAs eluted by DDX6 IP but rather could function as the machinery to harness these miRNAs. Computational analyses were used to predict the mRNA targets of miRNAs in DDX6-IP proteins (Fig 4F, S4 Table). Computational analyses were used to predict the mRNA targets of miRNAs in DDX6-IP proteins (Fig 4F, S4 Table). These mRNA targets included signaling pathways (MAPK, mTOR, PI3K-Akt, and TGF-β signaling pathways; the TGF-β pathway is central in MET), which are important for iPS reprogramming [31]. There were no significant differences in microarray analysis between siNeg- and siRNY1-treated TIG-1 fibroblasts, except for *BMPR2* and *PPP2CA* genes in the TGF-β signaling pathway (Fig 4G). Subsequently, we focused on the relationship between miRNA and the TGF-β signaling pathway. We selected the high expression of miR-302b-3p, which is strongly induced by OSKM and promotes the reprogramming of human fibroblasts to iPSCs. miR-302b-3p targets the 3′ untranslated region (3′ UTR) of *TGFβR2*, a key regulator of EMT that inhibits iPS reprogramming [32]. We examined whether *RNY1* regulates RNA metabolism by miR-302b-3p-mediated RNA decay using an *in vitro* luminescent analysis, using the pmirGLO Dual-Luciferase miRNA Target Expression Vector (S6 Fig). Luciferase activity was significantly lower in control fibroblasts than in *RNY1-*knockdown fibroblasts, suggesting that *RNY1-*knockdown inhibited miR-302b-3p-mediated RNA decay for *TGFβR2* (Fig 4H).

We analyzed protein expression differences in the cytoplasm between *RNY1-*knockdown and control cells using an iTRAQ proteomics analysis. In total, 722 proteins were significantly expressed in *RNY1-*knockdown fibroblasts (Parts A and B of S5 Fig, S5 Table). Nine were categorized as mesenchymal proteins, ECMs, and cell matrix adhesion proteins, in agreement with previous results [20]. These proteins are closely related to MET. Next, we checked the expression of MET-related genes within 72 h of OSKM induction (Part C of S5 Fig). *RNY1-*knockdown in fibroblasts revealed that many genes support mesenchymal properties, as evidenced by increases in *SLUG*, *ZEB1*, and *ZEB2*.

### The RO60/DDX6 axis regulates RNA decay for pre-existing RNAs at the early reprogramming stage

**Fig 5:**
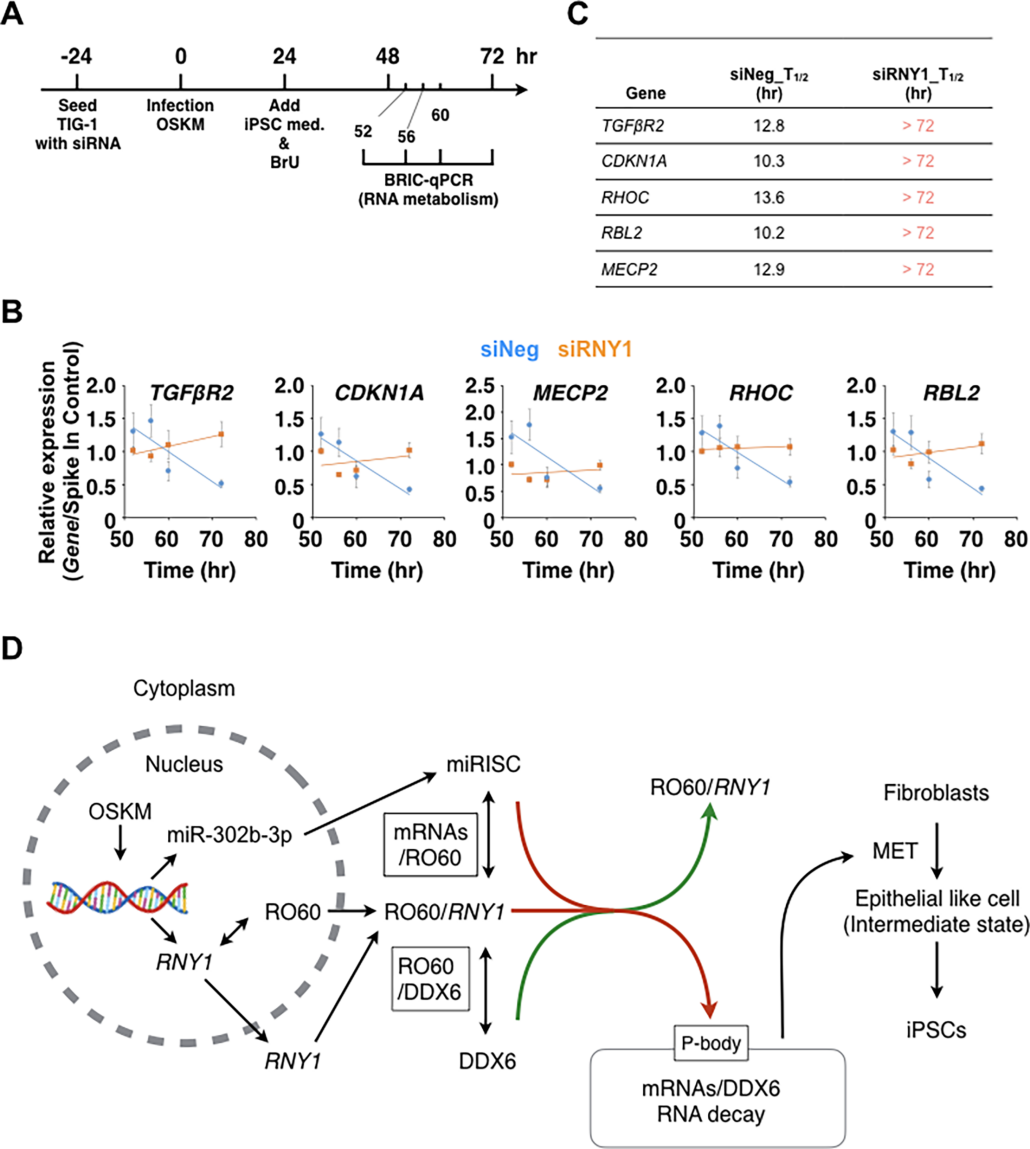
*RNY1* is essential for RNA decay via the regulation of DDX6. (A) Schematic representation of the early iPS reprogramming analysis using the BRIQ-qPCR method. (B) BrU-RNA degradation in OSKM- and siRNA-treated TIG-1 fibroblasts. Individual RNA expression levels were normalized to the Spike-In control. Data are presented as the mean ± SEM. (C) Table of T_1/2_ of OSKM- and siRNA-treated TIG-1 fibroblasts. (D) Illustration of the proposed model to explain the molecular mechanism for RNA decay at the early reprogramming stage.

To determine whether RNA decay or translational repression occurs in miRNA-mediated RNA silencing, we measured the amount and stability of miR-302b-3p-targeted RNA expression using BRIC-qPCR [33] (Fig 5A). From 52 to 72 h, all RNAs in siRNY1-treated fibroblasts were strongly stabilized, whereas all RNAs in control cells rapidly decreased (Fig 5B). We calculated the mRNA half-life (T_1/2_) for each mRNA sample (Fig 5C). Those of all RNA molecules in siRNY1-treated fibroblasts were longer than 72 h, whereas those for RNA molecules in control cells were less than 20 h. These results indicated that miR-302b-3p-targeted RNAs undergo a marked amount of degradation during this period and that *RNY1-*knockdown can inhibit the RNA decay process.

Computational protein–protein interactions in the presence of *RNY1* were modeled for RO60 and DDX6 [34]. The expected hydrogen bonds of Lys140 from hRO60 and Leu332 from DDX6 or Thr260 from hRO60 and Asn335 from DDX6, if the hydroxyl group of Thr260 rotates toward Asn335, are quite close to the interface for RecA2 of DDX6 and the CNOT1 MIF4G domain [35, 36] (S7 Fig). CNOT1 is a key component of the deadenylase complex, termed CCR4-NOT, which is a major trigger of miRNA-mediated deadenylation and mRNA decay [37]. Based on the model, there is one possibility of DDX6 possessing competitive ligands for RO60 and CNOT1. These models provided evidence for the possibility that the access of the 3′ deadenylation complex, which contains CNOT1, to RO60/*RNY1*/DDX6 removes DDX6 from the complex, along with the 3ʹ deadenylation complex containing mRNA.

We proposed a model describing the molecular mechanism for RNA decay at the early reprogramming stage (Fig 5D). After RO60 binds to *RNY1* in the nucleus, which conceals a nuclear accumulation signal, the complex is transferred to the cytoplasm. Abundant DDX6 assembles R060/*RNY1* to a fragile trimer. In the early reprogramming stage, nascent miRISCs provide a shortened and unstructured 3′ tail to RO60 and simultaneously to a competitor, CNOT1, for RO60/DDX6 binding. The RO60/*RNY1* complex is dissociated from the DDX6/miRISC complex, which forms a P-body. The DDX6/miRISC complexes degrade miR-302b-3p-targeted pre-existing RNAs related to mesenchymal properties, which leads to MET, an essential step in early reprogramming.

## Discussion

The newly transcribed *RNY1* is transferred to the cytoplasm in the form of an export receptor complex containing RO60 and RanGTPase, and the latter is then imported back into the nucleus after delivery to the cytoplasm [38]. Nuclear *RNY1* plays an essential role in the establishment and initiation of DNA replication forks [39]. Most proposed roles of *RNY*s involve nuclear functions, such as DNA replication or small RNA quality control. In this study, during the early reprogramming stage, *RNY1*, which is strongly induced by OSKM, transported RO60, after *RNY1* masked it in the central cavity of RO60, from the nucleus to the cytoplasm. The results from this study demonstrated that *RNY1* has a cytoplasmic function in RNA metabolism, specifically RNA decay.

A model based on the homology of RYPER posits that RO60 could function as an RNA chaperone to transfer mRNAs from RO60 to DDX6. If deadenylating mRNAs with miRISC were recruited to the ring of RO60, *RNY1* might relocate to the outer ring surface to open the central cavity of RO60, as demonstrated in RYPER. The RO60/*RNY1*/DDX6 complex might offer a domain with high affinity to RNAs. Our results demonstrated that DDX6 binds directly to RO60. In the early stage of reprogramming, DDX6 dissociated from RO60 in the presence of *RNY1* when an aggregate containing DDX6 began to form, whereas in *RNY1-*knockdown cells, DDX6 remained bound to RO60 (Fig 3D). DDX6 directly associates with the CCR4-NOT complex via an N-terminal fragment of CNOT1 [14, 40] and binds to 4E-T to recognize the 5′-end cap, consequently forming a bridge between the decapping and deadenylation (miRISC) machineries [41]. The bent mRNA seemed to recruit more DDX6, which possessed sequence-independent binding activity [29]. DDX6 in relaxed and extended mRNPs might have different affinities to RO60, leading to the decoupling of RO60/*RNY1*/DDX6, as it dissociates Edc3 and Pat1 following the association between DDX6 and CNOT1 [36]. CNOT1 contacts RecA2 of DDX6 via the final HEAT repeats, which RO60 also possesses, suggesting that CNOT1 and RO60 compete for binding to DDX6. After dissociation from the RNP, RO60/*RNY1* might be reused to recruit and remove 3′-shortened mRNA from the cytoplasm. Although the mechanism behind the dissociation is unclear, the released RNPs sequentially aggregate to create P-bodies, thus decreasing mesenchymal mRNA. The depletion of DDX6 completely abrogates fibroblast reprogramming to iPSCs, suggesting that DDX6 is indispensable in this process. *RNY1* is a critical regulator of DDX6; siRNY1 inhibits the dissociation of DDX6 from RO60 complexes, the formation of P-bodies, and reprogramming. A model for the RO60/ DDX6 axis is shown in Fig 5D.

Life starts from the union of two differentiated cells, an egg and a spermatozoon, and the zygote temporally gains totipotency. The change in cellular state is defined as the maternal-to-zygotic transition (MZT) [42] and is characterized by maternal mRNA/protein clearance [43] and zygotic genome activation [44]. Cellular reprogramming for pluripotency *in vitro* might exhibit parallels to the MZT [45]. Mutations of *smaug* [46] in *Drosophila* and Zfp36l2 in mice [47], both of which are involved in mRNA degradation, result in failure to complete the MZT, leading to developmental arrest. For *in vitro* fertilization-derived human embryos, poor development has been attributed to failure in the decay of maternal transcripts and not to inadequate zygotic gene activation [48]. During the MZT, microRNAs contribute to mRNA decay, as exemplified by *miR-430/427/302* in *Xenopus* [49] and *miR-309* in *Drosophila* [50]. On the other hand, iPSC generation was shown to be significantly improved by the addition of *miR-302/294* to OSKM-expressing cells. A cocktail of *miR-200c/302/369* alone for reprogramming surpassed the efficacy of the basic OSKM protocol [51]. In this study, *miR-302* cooperated with DDX6 for mRNA decay in P-bodies in order to eliminate not only pre-existing transcripts but also structural transcripts. Cellular transitions occur in a variety of situations, such as cancer metastasis, wherein EMT initially provides cancer cells mobility and MET enables tumor formation in new locations. As the persistence of pre-existing transcripts blocks reprogramming via the RO60/ DDX6 axis, the molecular components in the axis could be a target for modulating cancer metastasis. In particular, RO60 has been extensively investigated with respect to autoimmunity and intracellular sensing, and the open/closed system of the central hole in the doughnut-like steric structure is a putative target.

## Materials and methods

### Cell culture and iPSC induction

TIG-1, human fetal lung-derived fibroblasts, were cultured in Dulbecco's modified Eagle's medium (DMEM; Wako Pure Chemical Industries, Ltd., Osaka, Japan) supplemented with 10% fetal bovine serum (FBS, Life Technologies, Carlsbad, CA, USA) (Growth medium) at 37°C under 5% CO_2_. For iPSC induction, TIG-1 cells (1 × 10^5^ per well in 6 well plate) were seeded and/or transfected with Silencer^®^ Select siRNA for the negative control (AM4611), RNY1 #1 (n267575), or RNY1 #2 (s530828) using RNAiMAX Transfection Reagent (Thermo Fisher Scientific Inc., Waltham, MA, USA) according to the manufacturer’s recommendations. On the next day, cells were reprogrammed using CytoTune version 1.0 or 2.0. (ID Pharma Co., Ltd., Ibaraki, Japan) 48 h after infection, medium was replaced with iPSC culture medium, Knockout DMEM, 20% Knockout serum replacement (KSR), GlutaMax, non-essential amino acids, pyruvic acids, and 20 ng/ml basic fibroblast growth factor (bFGF; Thermo Fisher Scientific Inc.). Cells were observed and images were captured using an Olympus IX71 inverted microscope (Tokyo, Japan).

For the over-expression assay, full-length *RNY1* was engineered by PCR. The PCR-amplified *RNY1* gene was placed between *Hin*dIII and *Eco*RV restriction enzyme sites on a pcDNA3.1 vector (kindly provided by M. Toyoda, Tokyo Metropolitan Institute of Gerontology, Tokyo, Japan). pcDNA3.1 without *RNY1* served as a control.

### Cell counting

Cell number and cell viability were measured using the automatic cell counter ADAM (Digital Bio, Seoul, Korea), according to the manufacturer’s recommendations.

### Alkaline phosphatase-positive colony counting

After Sendai virus infection, infected TIG-1 cells were formed embryonic stem (ES) cell-like colonies. These colonies were stained using the BCIP/NBT Substrate System (Agilent Technologies, Santa Clara, CA, USA), according to the manufacturer's recommendations. Alkaline phosphatase (AP)-positive colonies were counting using ImageJ (National Institutes of Health, Bethesda, MD, USA).

### RNA and protein extraction

Cytoplasmic RNAs and proteins were extracted. The cytoplasmic fraction was separated from whole cell pellets using cell lysis buffer, including 10 mM Tris-HCl (pH 7.5), 0.1 M NaCl, 0.5% NP-40, RNaseOut (Thermo Fisher Scientific Inc.), and Proteinase Inhibitor Cocktail (Sigma, St. Louis, MO, USA). Cells were reacted in cell lysis buffer for 15 min at 4°C. Then, cells were centrifuged at 12,000 × *g* for 15 min at 4°C. After centrifugation, the supernatant was collected as the cytoplasmic fraction. To extract cytoplasmic RNAs, a cytoplasmic protein fraction was supplemented with 4 parts TRIzol^®^ RNA Isolation Reagent (Thermo Fisher Scientific Inc.). Precipitation of the nuclear fraction was performed by adding TRIzol^®^ RNA Isolation Reagents, followed by homogenization using a 25-gauge syringe. RNAs from whole cells, cytoplasm, and nuclear fractions were extracted using TRIzol^®^ RNA Isolation Reagent and the Direct-zol™ RNA MiniPrep Kit (Zymo Research, Irvine, CA, USA) according to the manufacturer’s recommendations.

### Quantitative reverse transcriptase polymerase chain reaction (qPCR)

For qPCR, 100 ng of total RNA was reverse-transcribed using the PrimeScript RT reagent Kit (Takara Bio Inc., Shiga, Japan) and KAPA SYBR FAST qPCR Kit Master Mix (2×) Universal (KAPA BIOSYSTEMS, Boston, MA, USA) according to the manufacturers’ recommendations, and MIQE guideline [52]. qPCR was performed using the Thermal Cycler Dice Real Time System (Takara Bio Inc.) or CFX Connect Real-Time PCR Detection System (Bio-Rad Laboratories, Inc., CA, USA). All reactions were performed in triplicate. An lncRNA analysis was performed using the LncProfiler qPCR Array Kit (System Biosciences, Inc., Palo Alto, CA, USA), according to the manufacturer’s recommendations. For qPCR, 10 ng of total RNAs was reverse-transcribed using TaqMan probes (Thermo Fisher Scientific Inc.), according to the manufacturer’s recommendations. To perform ChIP-qPCR, genomic DNAs from formamide-fixed cells were analyzed using Chromatin Immunoprecipitation (ChIP) Kits (Takara Bio Inc.) according to the manufacture’s recommendations. The primers and antibodies are listed in S6 Table.

### Computational analysis

The sequence of human *RNY1* was obtained from the NCBI database (NR_004391.1). The secondary structure of *RNY1* was predicted using the RNAfold web server (http://rna.tbi.univie.ac.at/cgi-bin/RNAfold.cgi), which shows secondary structures of single-stranded RNA or DNA sequences. Structure modeling of hRO60 was obtained by using HHPred server (https://toolkit.tuebingen.mpg.de/hhpred) [53, 54]. Crystal structure of RO60 from *Xenopus* (Protein Data Bank (PDB) ID: 1YVP, sequence identity: 78%) was used as a template structure and homology modeling was performed with HHPred’s default settings. For complex of hRO60 and *RNY1*, *RNY1* chain (chains C and D) from crystal structure of *Xenopus* RO60 (PDB ID: 1YVP) was placed by superimposing *Xenopus* RO60 and modeled hRO60. The modeled hRO60 structure and *RNY1* are shown in Fig 1D.

Interaction between hRO60 and DDX6 was modeled by the protein-protein docking software MEGADOCK (version 4.0.2)[34, 55]. Modeled structure of hRO60 as described above and crystal structure of human DDX6 (PDB ID: 4CT5, chain A) were used for building complex models. As the setting of MEGADOCK, 6° rotation interval and 5,400 solutions were used (command line options for ‘–N 5400 –D’). Then, 850 solutions including the residue-residue interactions same as CNOT1 MIF4G domain were selected with reference to the co-crystal structure of CNOT1 and DDX6 (PDB ID: 4CT4). Next, we manually selected 21 solutions contains interactions with HEAT repeats interaction and no heavy collision with superimposed *RNY1*. Finally, we selected and showed two solutions as S7 Fig.

Global gene expression analysis

### Microarray

A gene expression analysis was performed using the SurePrint G3 Human GE Microarray 8×60K Ver. 2.0 (Agilent Technologies). Raw data were normalized and analyzed using MeV: MultiExperiment Viewer (http://www.tm4.org/mev.html). Gene Ontology (GO) and Kyoto Encyclopedia of Genes and Genomes (KEGG) pathway enrichment results were evaluated statistically following the instructions provided by the Database for Annotation, Visualization and Integrated Discovery (DAVID) 6.7[56]. Gene expression microarray data have been submitted to GEO (Gene Expression Omnibus) under accession number GSE118887.

### RNA immunoprecipitation, RIP for the miRNA-nCounter analysis

RIP was performed following a previously described procedure, with modifications. Samples were pre-cleared with BcMag™ Protein A/G Magnetic Beads (Bioclone Inc., San Diego, CA, USA) for 2 h at 4°C, followed by the addition of 10 μg of the antibody. Protein G beads (20 μl) were added after 2 h of incubation and left for another 1 h at 4°C. Beads were washed once with 1 ml of binding buffer (50 mM HEPES/0.5% triton/25 mM MgCl_2_/5 mM CaCl_2_/20 mM EDTA), once with FA500 (50 mM HEPES/500 mM NaCL/1 mM EDTA/1% triton/0.1% Na deoxycholate), once with LiCl buffer (10 mM Tris/250 mM LiCl/1% triton/0.5% Na deoxycholate/1 mM EDTA), and once with TES (10 mM Tris/10 mM NaCl/1 mM EDTA). Immunoprecipitates were eluted with 75 μl of RIP elution buffer (100 mM Tris pH 7.8/10 mM EDTA/1% SDS). NaCl was adjusted to 200 mM and the samples were treated with 20 μg of proteinase K for 1 h at 42°C and 1 h at 65°C. RNA was extracted with TRIzol^®^ RNA Isolation Reagents.

nCounter

Immunoprecipitated miRNAs or stem cell-related genes were analyzed using Nanostrings nCounter probe sets of nCounter Human miRNA Assay Kits or the nCounter Virtual Stem Cell Gene Set (NanoString® Technologies, Inc., Seattle, WA, USA). Raw data were normalized and analyzed using nSolverAnalysis Software (NanoString® Technologies, Inc.) and MeV. The miRNAs and gene expression nCounter data have been submitted to GEO (Gene Expression Omnibus) under accession number GSE118888.

### Immunocytochemistry

Cells were fixed with 4% paraformaldehyde (Wako Pure Chemical Industries, Ltd., Saitama, Japan) for 10 min at 24°C, and reacted with 0.1% TritonX-100 (Sigma-Aldrich) and 5% of goat normal serum (Agilent Technologies) in PBS (Wako Pure Chemical Industries, Ltd.) for 10 min. Cells were then incubated overnight with each primary antibody (S6 Table) in PBS at 4°C. They were then incubated at 24°C with the secondary antibody for each primary antibody conjugated with Alexa Fluorescent dye (1:300 dilution, Thermo Fisher Scientific Inc.). The nuclei were counterstained with 4, 6-diamidino-2-phenylindole (DAPI) (Wako Pure Chemical Industries, Ltd.) for 45 min. To prevent fading, cells were then mounted in DakoCytomation Fluorescent Mounting Medium (Agilent Technologies). Samples were observed and images were captured with an Olympus IX71 inverted microscope (Tokyo, Japan) or a Keyence BZ-X700 digital microscope (Osaka, Japan). F-actin was discerned by staining with CytoPainter Phalloidin-iFluor 594 Reagent (ab176757, Abcam).

### Western blotting

Cytoplasmic protein (50 μg) was dissolved in Laemmli's buffer, boiled for 10 min, electrophoresed on a 10% SDS polyacrylamide gel, and electroblotted onto a PVDF transfer membrane (Millipore, Billerica, MA, USA). The membrane was blocked with PBS containing 5% skim milk and 0.05% Tween 20 and then incubated for 1 h with each antibody (diluted to 1:500 with blocking buffer). After washing, the membrane was incubated with 1:5000 diluted horseradish peroxidase (HRP)-conjugated Donkey anti-Rabbit IgG or HRP-conjugated Donkey anti-Mouse IgG (GE Healthcare, Little Chalfont, UK) in Blocking Buffer. Subsequently, the blots were developed using the ECL Detection Kit (GE Healthcare) and protein bands were visualized using the VersaDoc System (Bio-Rad Laboratories, Inc., Hercules, CA, USA).

### Immunoprecipitation and immunoblot analyses

Magnetic beads, Magnosphere^TM^ MS300/Carboxyl (JSL life Sciences Corp., Ibaraki, Japan), for immunoprecipitation were prepared according to the manufacturer’s recommendations. Protein lysates were incubated with anti-RO60 or anti-DDX6 antibody-conjugated magnetic beads for 2 hr at 4°C. The immune complexes were analyzed.

### Nano-flow liquid chromatography-ion trap mass spectrometry (LC-MS/MS)

Gel bands were excised and soaked in 50 mM Tris-HCl, pH 8.0, containing 50% acetonitrile for 30 min. The gel was dried in a Speed-Vac (Savant, Hyannis, MA, USA) and incubated in 50 mM Tris-HCl, pH 8.0 containing 125–250 ng of modified trypsin (Roche Diagnostics, Mannheim, Germany) or chymotrypsin (Roche Diagnostics) at 37°C for 6–20 h. The digests were extracted from the gel twice with 100 μl of 0.1% TFA containing 60% acetonitrile. These two extracts were combined, evaporated in a Speed-Vac, and stored at -80°C until use.

The sample was resuspended in 0.1% formic acid containing 2% acetonitrile and introduced into a nano-flow HPLC system, DiNa-fitted with an automatic sampler (KYA Technology Corporation, Tokyo, Japan). The packed nano-capillary column NTCC-360/75-3-123 (0.075 mm I.D. × 125 mm L, particle diameter 3 μm, Nikkyo Technos Co., Ltd., Tokyo, Japan) was used at a flow rate of 300 nl/min with a 2–80% linear gradient of acetonitrile for 60 min. Eluted peptides were directly detected with an ion trap mass spectrometer, Velos Pro (Thermo Fisher Scientific Inc.) at a spray voltage of 1.9 kV and a collision energy of 35%. The mass acquisition method consisted of one full MS survey scan followed by an MS/MS scan of the most abundant precursor ions from the survey scan. Dynamic exclusion for MS/MS was set to 30 s. An MS scan range of 400–2000 m/z was employed in the positive ion mode, followed by data-dependent MS/MS using the CID or HCD operating mode on the top 10 ions in order of abundance. The data were analyzed using Proteome Discoverer (Thermo Fisher Scientific Inc.), Mascot (Matrix Science Inc., Boston, MA, USA), and Scaffold (Proteome Software, Inc., Portland, OR, USA). Swiss-Prot and GenBank databases were used.

### Protein-protein binding kinetics using the BLItz instrument

Recombinant human RO60 (rRO60, ab73786) and Recombinant GST-tagged human DDX6 (rDDX6, ab114574) proteins were purchased from Abcam (Cambridge, MA, USA). Protein interactions were examined using the BLItz system from Pall ForteBio LLC (Fremont, CA, USA). All kinetic experiments were carried out at 25°C. An antibody against RO60 at 50 ng/ml was captured on a Protein-A biosensor. Then, 50 ng/ml rRO60 in 10 mM Tris-HCl (pH 8.0) and 150 mM NaCl was bound to the anti-RO60-reacted Protein-A biosensor. Furthermore, 25 ng/ml rDDX6 was bound to rRO60 for 2 min and allowed to dissociate in the same Tris-HCl buffer. Dissociation was monitored for 2 min. Kinetic data were analyzed using BLItz^TM^ Pro.

### Lentivirus transduction for DDX6 knockout TIG-1 fibroblasts

To knock out DDX6 using clustered regularly interspaced short palindromic repeats (CRISPR)/Cas9 technology, sgRNAs targeting tdTomato (negative control), DDX6 exon-1, and RO60 exon-2 were cloned into a Cas9-expressing lentiviral transfer vector (lentiCRISPRv2, Cat No. 52961; Addgene, Cambridge, MA, USA) following the methods of the Feng Zhang laboratory [57]. The following oligonucleotides from the sense strands were used for gRNAs targeting tdTomato (Forward, 5’- CACCGCCCCGCGACGGCGTGCTGAA-3’; Reverse, 5’- AAACTTCAGCACGCCGTCGCGGGGC-3’), DDX6 exon-1 (Forward, 5’-CACCGTATAACAGGGTTCTCTGTTC-3’; Reverse, 5’-AAACGAACAGAGAACCCTGTTATAC-3’) and RO60 exon-2 (Forward, 5’- CACCGTCCGGAAGGCTATAGCGGAC-3’; Reverse, 5’- AAACGTCCGCTATAGCCTTCCGGAC-3’). These oligonucleotides were ligated into lentiCRISPRv2 according to Feng Zhang laboratory protocol (lentiCRISPRv2 and lentiGuide oligo cloning protocol)[57]. To prepare lentiviruses for tdTomato, *DDX6*, and *RO60* gene disruption, lentiCRISPRv2–sgRNA tdTomato, DDX6, and RO60 transfer plasmids were co-transfected with the packaging plasmids pMD2.G and psPAX2 (Addgene plasmids 12259 and 12260). For viral transduction for the gene disruptions of tdTomato, DDX6, and RO60, 1 × 10^5^ TIG-1 fibroblasts were incubated with the 0.2 μm-filtered lentivirus-containing supernatant. Three days after infection, puromycin was added to screen sgRNA/Cas9-positive cells. Two weeks later, the cell culture was expanded to three 35-mm dishes. In order to assess the efficiency of sgRNA-guided Cas9 cutting in the *DDX6* and *RO60* genomic sequences, protein deletions were confirmed by western blotting.

### Luciferase reporter assays for miRNA analysis

The 2,535-bp sequence of the *TGFβR2* 3′ UTR contains the predicted hsa-miR-302b-3p-binding sites [32]. The DNA fragments were amplified and digested with *Nhe*I and *Sal*I-conjugated primers. The resulting fragments were subcloned into these sites of the pmirGLO Dual-Luciferase miRNA Target Expression Vector (Promega Corp., Madison, WI, USA), referred to as pmirGLO-TGFβR2. *TGFβR2* 3′ UTR sites were amplified by Platinum^®^ Taq DNA Polymerase, High Fidelity (Thermo Fisher Scientific). The pmirGLO and siNeg or siRNY1 transfections were carried out with Lipofectamine 2000 (Thermo Fisher Scientific) according to previous reports [58–60]. On the following day, the transfected cells were reprogrammed using CytoTune version 2.0 (OSKM). Two or three days after OSKM induction, the measurements of firefly luciferase and *Renilla* luciferase were performed stepwise using the Dual-Glo luciferase assay system and a GloMax^®^ 20/20 Luminometer (Promega Corp.). d2 and d3 indicate 2 or 3 days after OSKM induction, respectively.

### RNA decay analysis in iPSC reprogramming

RNA decay was analyzed using the RiboCluster Profiler™ BRIC Kit (Medical & Biological Laboratories Co., Ltd., Nagoya, Japan) according to the manufacturer’s instructions. In brief, siRNA-transfected cells were incubated at 37°C in Growth medium with CytoTunes for 24 h in a humidified incubator with 5% CO_2_. At 24 h after infection, Growth medium was replaced with medium containing 150 μM 5′-bromo-uridine (BrU) for 24 h. After replacing BrU-containing medium with BrU-free growth medium, cells were harvested at 52, 56, 60, and 72 h. Total RNA was isolated using TRIzol^®^ RNA Isolation Reagents. Total RNAs were denatured by heating at 80°C for 1 min and then added to anti-BrdU mAb-conjugated beads containing 2 μg of anti-BrU mAb (clone 2B1, MBL). The mixture was incubated at room temperature for 1 h with rotation. Beads were washed four times with 0.1% BSA in PBS. Total RNAs were isolated using TRIzol^®^ RNA Isolation Reagents and used for qRT-PCR. RNA half-lives were calculated using Microsoft Excel software.

### iTRAQ labeling, sample cleaning, and desalting

The proteins extracted from the retinas were labeled with isobaric Tags for Relative and Absolute Quantification (iTRAQ) Reagents - 8plex Applications Kit (AB Sciex, Foster City, CA, USA) according to the manufacture’s instruction. Briefly, 50 μg of the extracted proteins were subsequently denatured and reduced, alkylated, and digested by trypsin at 37°C overnight. Digested samples were labeled with iTRAQ reagents at room temperature, and labeled peptides were pooled. A cation exchange cartridge system (AB Sciex) was used to remove the reducing reagent, SDS, excess iTRAQ reagents, undigested proteins, and trypsin in the labeled sample mixture to prevent interference with the LC/MS/MS analysis. The sample mixture was loaded onto the cation exchange cartridge. After washing with 8 column volumes of Cation Exchange Buffer-Load, peptides were eluted using Cation Exchange Buffer-Elute at various concentrations (17.5, 35, 52.5, 70, 105, 140, 175, and 350 mM). Eluted samples were desalted using a Sep-Pak C18 Plus Light Cartridge (Waters Corporation, Milford, MA, USA). Each eluted sample was loaded onto the Sep-Pak C18 Plus Light Cartridge. After washing with buffer (0.1% formic acid (FA)), peptides were desalted and eluted with elution buffer (70% ACN, 0.1% FA). Each eluted sample was dried and supplemented with 30 μl of 0.1% FA.

### NanoLC-MS/MS analysis

The analysis was performed using a 5600 TripleTOF (AB Sciex) interfaced with a DiNa Direct Nano—flow LC system (KYA Technologies). The eluent from each injection of desalted samples was subjected directly to the trap column and sequentially to the analytical column using a gradient of 0–45% solvent B in solvent A over 140 min [solvent A: 0.1% FA, 2% ACN; solvent B: 0.1% FA, 80% ACN] and 45–100% solvent B for 15 min at a flow rate 300 nl/minute. The RP column eluent was analyzed using a TripleTOF 5600 (AB Sciex). For standard data-dependent analyses, the mass spectrometer was operated in a manner where a 250-ms survey scan (TOF-MS) was collected, from which the top 20 ions were selected for automated MS/MS in subsequent experiments, where each MS/MS event consisted of a 50-ms scan.

iTRAQ Data Analysis

Relative abundance quantitation and peptide and protein identification were performed using ProteinPilot 4.5 (AB Sciex). Each MS/MS spectrum was searched for homologs in *Homo sapiens* against the NCBI database. The unused protein score is the ProteinPilot measurement of protein identification confidence, taking into account all peptide evidence for a protein, excluding any evidence that is better explained by a higher-ranking protein. Relative quantification of proteins in the case of iTRAQ was performed on the MS/MS scans and was calculated as the ratio of the areas, which were the masses of the tags that correspond to the iTRAQ reagents.

### Statistical analysis

Results are expressed as means ± standard error (SE). The statistical significance of differences among groups was evaluated using *t*-tests, Standard Bonferroni correction (*p* = 0.01) (TIGR-TM4-MEV, and Prism 6 software, GraphPad Prism Software Inc., San Diego, CA), and *p* < 0.05 was considered significant.

## Supporting information

S1 Fig Relationship between DDX6 and iPS reprogramming. (A) Immunoblotting of RO60 and DDX6 antibodies in TIG-1 fibroblast whole lysates and recombinant RO60 (60 kDa) and DDX6 (79 kDa). Recombinant DDX6 included GST-tag. Molar ratios of RO60 and DDX6 including TIG-1 whole proteins were calculated based on calibration curves using each recombinant protein and ImageJ. Each protein was standardized by the amount applied in the lane. (B) Immunoblotting of RO60 and DDX6 antibodies to TIG-1 fibroblast lysates from W: whole, N: nuclear, and C: cytoplasm fractions. (C) Growth curve of lentiCRISPR v2-treated TIG-1 fibroblasts. (D) Phase contrast micrograph images of OSKM-transduced TIG-1 fibroblasts control (cntrl), genomic disruption targeting RO60 (Cas9_RO60), DDX6 (Cas9_DDX6), and tdTomato (Cas9_Tom) as a negative control using the CRISPR/Cas9 system at Day 20.
(TIFF)

S2 Fig Cytoplasmic RNA and protein expression analysis. (A) RNA expression levels of OSKM- and siRNA-treated TIG-1 fibroblasts at Day 3. Total RNAs were collected from the cytoplasm. (B) Protein expression levels of OSKM- and siRNA-treated TIG-1 fibroblasts at Day 3 with RO60, DDX6, and GAPDH antibodies. Total proteins were collected from the cytoplasm. The raw blotting data are attached to S8 Fig. (C) Immunocytochemical results for DDX6 and OCT4 of TIG-1 fibroblasts at Day 3.
(TIFF)

S3 Fig Global gene expression analysis of iPS reprogramming on Day 3. (A) Heat map showing microarray analysis results of OSKM- and siRNA-treated TIG-1 fibroblasts (n = 2). (B) Genes were categorized based on biological processes using Gene Ontology (GO) annotations in siNeg > (Black bars) and siRNY1 > (White bars). There were significant differences between treatments. (C) Genes were categorized based on Kyoto Encyclopedia of Genes and Genomes (KEGG) pathway annotations in siNeg > (Black bars) and siRNY1 > (White bars). (D) Significant expression changes in siRNA-treated TIG-1 fibroblasts. Log_2_ ratios of RNAs with significant expression changes in both siNeg and siRNY1 on Day 3. Colored dots indicate groups with substantial changes (y > x + 0.5). RNA expression levels in siRNA- and OSKM-treated TIG-1 fibroblasts with significant changes were analyzed from Days 1 to 3. (E) Heat map showing microarray analysis results of untreated TIG-1 fibroblasts and OSKM- and siRNY1-treated TIG-1 fibroblasts. There were no significant differences between cells. Genes were categorized according to cellular component (CC, Black bar), biological process (BP, White bar), and molecular function (MF, Gray bar) based on GO terms.
We compared global gene expression in *RNY1-*knockdown and control TIG-1 fibroblasts on Day 3 during OSKM transduction using the Agilent Human Microarray Chip. In total, 281 annotated genes were specifically expressed in control fibroblasts, and 278 were specifically expressed in *RNY1-*knockdown fibroblasts (Part A of S3 Fig, S3 Table). These genes were categorized based on Gene Ontology (GO) annotations and Kyoto Encyclopedia of Genes and Genomes (KEGG) pathways (Parts B and C of S3 Fig, S3 Table). In the biological process category, genes specifically expressed in *RNY1-*knockdown fibroblasts on Day 3 were assigned to functional categories related to the cell cycle and cell division, and those in the control fibroblasts were assigned to functional categories related to RNA metabolism and cellular differentiation (Part B of S3 Fig). These results support the data indicating higher rates of cell growth in *RNY1-*knockdown fibroblasts than in control fibroblasts. The gene cluster related to RNA metabolism decreased in *RNY1-*knockdown fibroblasts, supporting our hypothesis that *RNY1* is involved in the metabolism of in mRNAs, especially the degradation or silencing of pre-existing mRNA pools. KEGG pathway analyses revealed that genes specifically expressed in control fibroblasts at the early reprogramming stage were functionally related to intracellular nucleic acid sensing and apoptosis (Part C of S3 Fig). The former category indicates that RNAs resembling viral RNA, such as RNAs transcribed by polymerase II, including *RNY1*, might be involved in the reprogramming process.
We mapped transcripts with significant expression differences between *RNY1-*knockdown and mock transfected fibroblasts at the early reprogramming stage in two-dimensional plots based on the log-transformed fold change. These RNA expression changes exhibited a strong correlation (*R*^2^ = 0.89), and some transcripts among the downregulated genes in the mock transfected fibroblasts were significantly higher than those in *RNY1-*knockdown, indicated as green and red dots in Part D of S3 Fig. Genes that were inhibited during cellular reprogramming included *SYNJ2* (involved in invadopodia formation in EMT for cancer metastasis), *MYO18A* and *NHS* (cytoskeleton-related genes), and *STEAP3* (mediates molecules downstream of *p53*, which inhibits reprogramming). After *RNY1-*knockdown during early reprogramming, some pre-existing RNA pools were preserved, and some pre-existing RNA degradation was prevented (Part D of S3 Fig). TP53 inhibition is critical for the progression of cellular reprogramming, and MYO18A and NHS confer basal mesenchymal properties, demonstrating that RNA decay via the RO60/ DDX6 axis is essential for reprogramming. Based on the similarities between cellular reprogramming and early development following fertilization, the carry-over of pre-existing RNA pools could be a hurdle in the process, similar to the maternal RNAs remaining in zygotes, which block the developmental process before embryonic genome activation [48]. Many genes did not exhibit significant differences between the *RNY1-*knockdown and control fibroblasts, such as chromosome and cell cycle genes (Part E of S3 Fig, S3 Table). Although many genes were inhibited during the reprogramming process, genes exhibiting reversed expression following *RNY1-*knockdown were also involved. The latter genes are likely silenced by different machineries. However, the observation that even partial carry-over of pre-existing RNAs strongly inhibited reprogramming suggests that RNA silencing is far more important than previously thought.
(TIFF)

S4 Fig Protein and miRNA analyses of DDX6-IP proteins or pre-IP proteins. (A) Immunoblotting for DDX6 and GAPDH of DDX6-IP protein from OSKM-transduced TIG-1 fibroblasts. (B) Volcano plot of global miRNA expression in whole TIG-1 fibroblast lysates using the nCounter system. (C) Heat map of miRNAs with *p-*values of less than 0.05 in whole proteins from TIG-1 fibroblasts using the nCounter system.
(TIFF)

S5 Fig Protein analysis in OSKM- and siRNA-treated TIG-1 fibroblasts at Day 3. (A) Global proteomics using the iTRAQ method. Red dots indicate MET-related proteins, e.g., mesenchymal, ECM, and cell-matrix adhesion proteins. (B) Proteins were categorized based on biological processes using Gene Ontology (GO) annotations. (C) mRNA expression in OSKM- and siRNA-treated TIG-1 fibroblasts from 0 to 72 h. Individual RNA expression levels were normalized to *GAPDH* expression levels. Data are presented as means ± SEM.
(TIFF)

S6 Fig Plasmid maps. (TIFF)

S7 Fig Computational protein–protein interactions analysis. The proposed two structure models of hRO60 and DDX6 complex. The molecular structures of hRO60, *RNY1* and DDX6 are colored as mocha, gray and aqua in (a), and green, gray and cyan in (b). The expected hydrogen bonds and their residues are represented as dashed lines and sticks, respectively, in close up views of the expected interaction between hRO60 and DDX6 in the presence of *RNY1*.
(TIFF)

S8 Fig Immunoblotting data. Raw blotting data in Fig 3D, and Part B of S2 Fig. The dotted-squares indicate the blotting bands used in each figure.
(TIFF)

S1 Table Global gene analysis during iPS reprogramming using the LncProfiler lncRNA qPCR array. (XLSX)

S2 Table Significantly expressed ncRNA at the early iPS reprogramming stage. (XLSX)

S3 Table Microarray analysis of OSKM- and siRNA-treated TIG-1 fibroblasts. (XLSX)

S4 Table MicroRNA analysis of DDX6-IP proteins. (XLSX)

S5 Table Global protein expression analysis using the iTRAQ method. (XLSX)

S6 Table Information of primers and antibodies. (XLSX)
