# Supplementary figures and images for: The DEAD-box RNA-binding protein DDX6 regulates parental RNA decay for cellular reprogramming to pluripotency
Source: PLoS One. 2018 Oct 1;13(10):e0203708. doi: 10.1371/journal.pone.0203708 (PMC6166933; doi:10.1371/journal.pone.0203708)

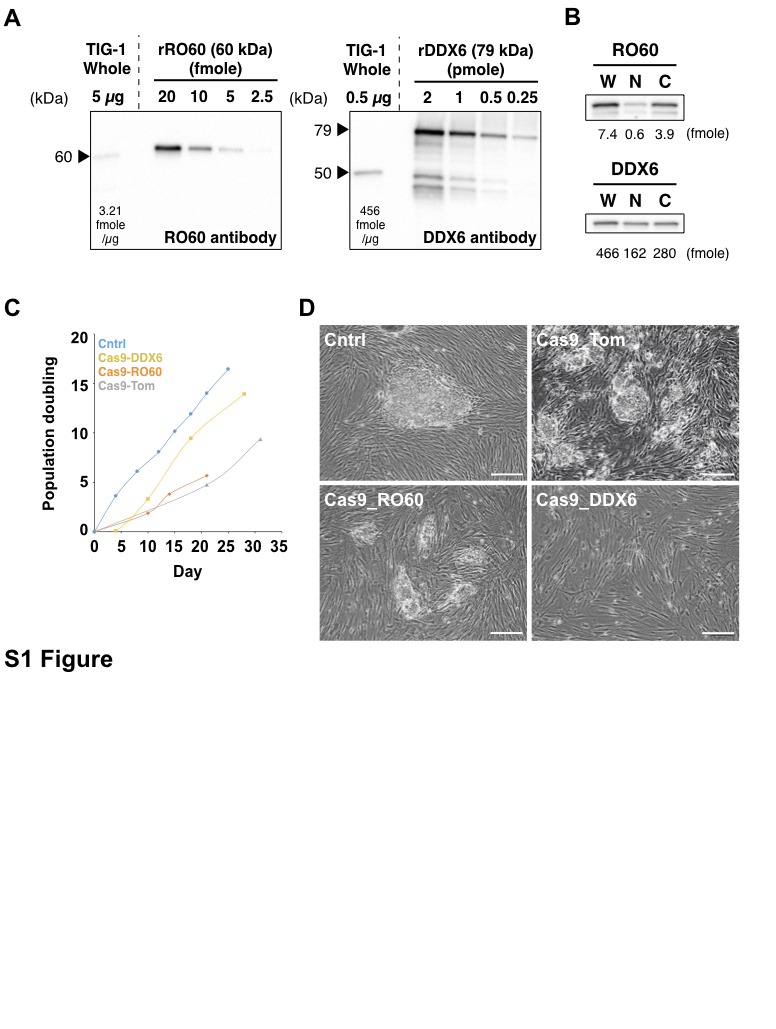

Supplement: S1 Fig — (A) Immunoblotting of RO60 and DDX6 antibodies in TIG-1 fibroblast whole lysates and recombinant RO60 (60 kDa) and DDX6 (79 kDa). Recombinant DDX6 included GST-tag. Molar ratios of RO60 and DDX6 including TIG-1 whole proteins were calculated based on calibration curves using each recombinant protein and ImageJ. Each protein was standardized by the amount applied in the lane. (B) Immunoblotting of RO60 and DDX6 antibodies to TIG-1 fibroblast lysates from W: whole, N: nuclear, and C: cytoplasm fractions. (C) Growth curve of lentiCRISPR v2-treated TIG-1 fibroblasts. (D) Phase contrast micrograph images of OSKM-transduced TIG-1 fibroblasts control (cntrl), genomic disruption targeting RO60 (Cas9_RO60), DDX6 (Cas9_DDX6), and tdTomato (Cas9_Tom) as a negative control using the CRISPR/Cas9 system at Day 20. (TIFF) [file pone.0203708.s001.tiff]

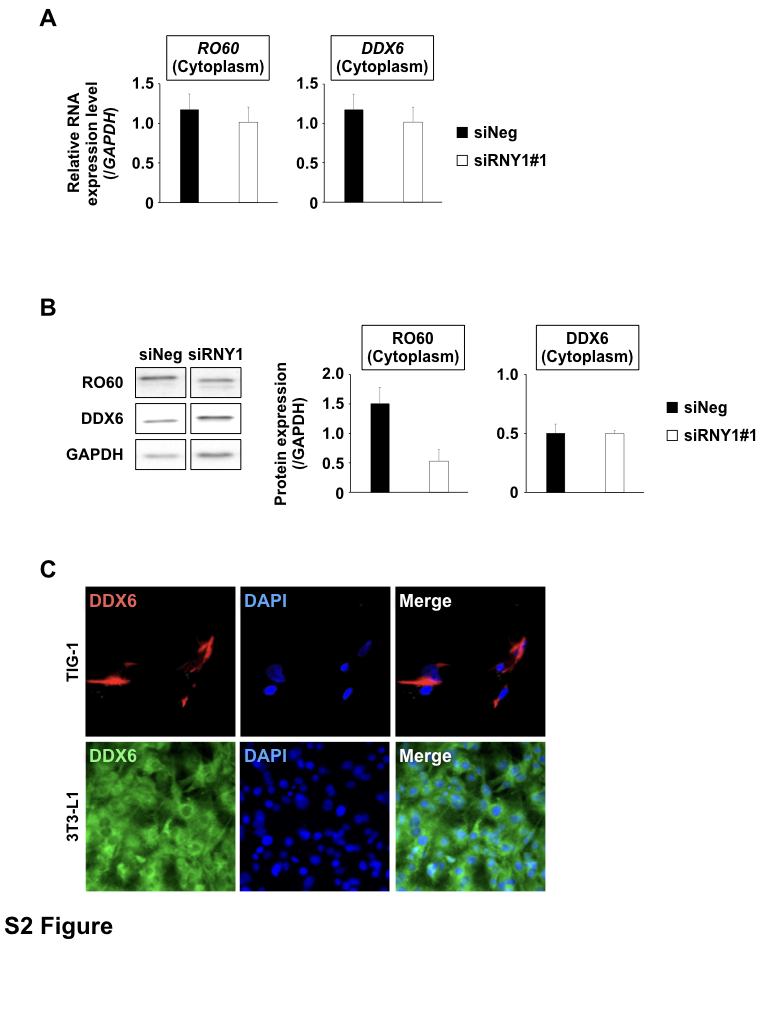

Supplement: S2 Fig — (A) RNA expression levels of OSKM- and siRNA-treated TIG-1 fibroblasts at Day 3. Total RNAs were collected from the cytoplasm. (B) Protein expression levels of OSKM- and siRNA-treated TIG-1 fibroblasts at Day 3 with RO60, DDX6, and GAPDH antibodies. Total proteins were collected from the cytoplasm. The raw blotting data are attached to S8 Fig. (C) Immunocytochemical results for DDX6 and OCT4 of TIG-1 fibroblasts at Day 3. (TIFF) [file pone.0203708.s002.tiff]

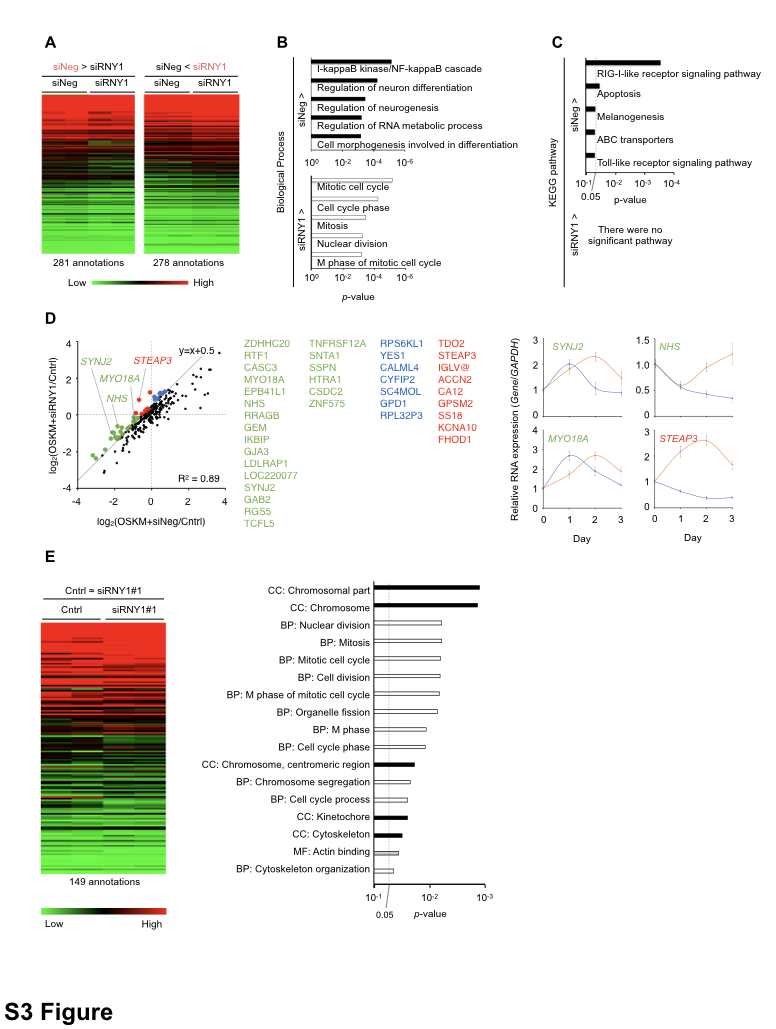

Supplement: S3 Fig — (A) Heat map showing microarray analysis results of OSKM- and siRNA-treated TIG-1 fibroblasts (n = 2). (B) Genes were categorized based on biological processes using Gene Ontology (GO) annotations in siNeg > (Black bars) and siRNY1 > (White bars). There were significant differences between treatments. (C) Genes were categorized based on Kyoto Encyclopedia of Genes and Genomes (KEGG) pathway annotations in siNeg > (Black bars) and siRNY1 > (White bars). (D) Significant expression changes in siRNA-treated TIG-1 fibroblasts. Log2 ratios of RNAs with significant expression changes in both siNeg and siRNY1 on Day 3. Colored dots indicate groups with substantial changes (y > x + 0.5). RNA expression levels in siRNA- and OSKM-treated TIG-1 fibroblasts with significant changes were analyzed from Days 1 to 3. (E) Heat map showing microarray analysis results of untreated TIG-1 fibroblasts and OSKM- and siRNY1-treated TIG-1 fibroblasts. There were no significant differences between cells. Genes were categorized according to cellular component (CC, Black bar), biological process (BP, White bar), and molecular function (MF, Gray bar) based on GO terms. We compared global gene expression in RNY1-knockdown and control TIG-1 fibroblasts on Day 3 during OSKM transduction using the Agilent Human Microarray Chip. In total, 281 annotated genes were specifically expressed in control fibroblasts, and 278 were specifically expressed in RNY1-knockdown fibroblasts (Part A of S3 Fig, S3 Table). These genes were categorized based on Gene Ontology (GO) annotations and Kyoto Encyclopedia of Genes and Genomes (KEGG) pathways (Parts B and C of S3 Fig, S3 Table). In the biological process category, genes specifically expressed in RNY1-knockdown fibroblasts on Day 3 were assigned to functional categories related to the cell cycle and cell division, and those in the control fibroblasts were assigned to functional categories related to RNA metabolism and cellular differentiation (Part B of S3 Fig). T [file pone.0203708.s003.tiff]

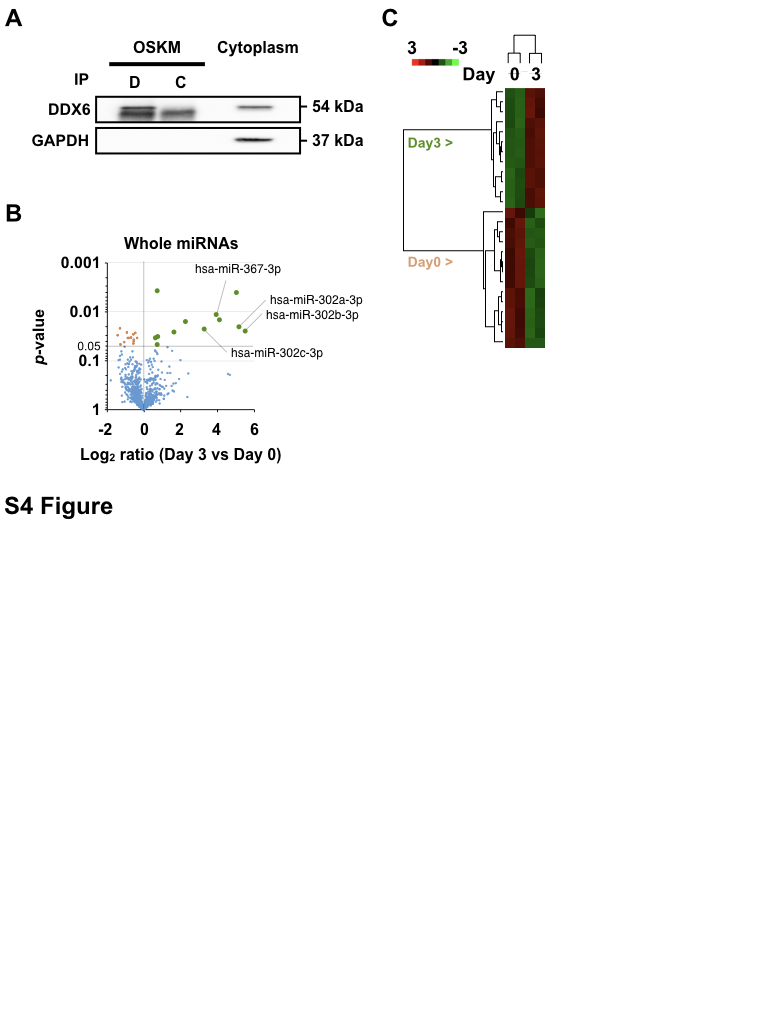

Supplement: S4 Fig — (A) Immunoblotting for DDX6 and GAPDH of DDX6-IP protein from OSKM-transduced TIG-1 fibroblasts. (B) Volcano plot of global miRNA expression in whole TIG-1 fibroblast lysates using the nCounter system. (C) Heat map of miRNAs with p-values of less than 0.05 in whole proteins from TIG-1 fibroblasts using the nCounter system. (TIFF) [file pone.0203708.s004.tiff]

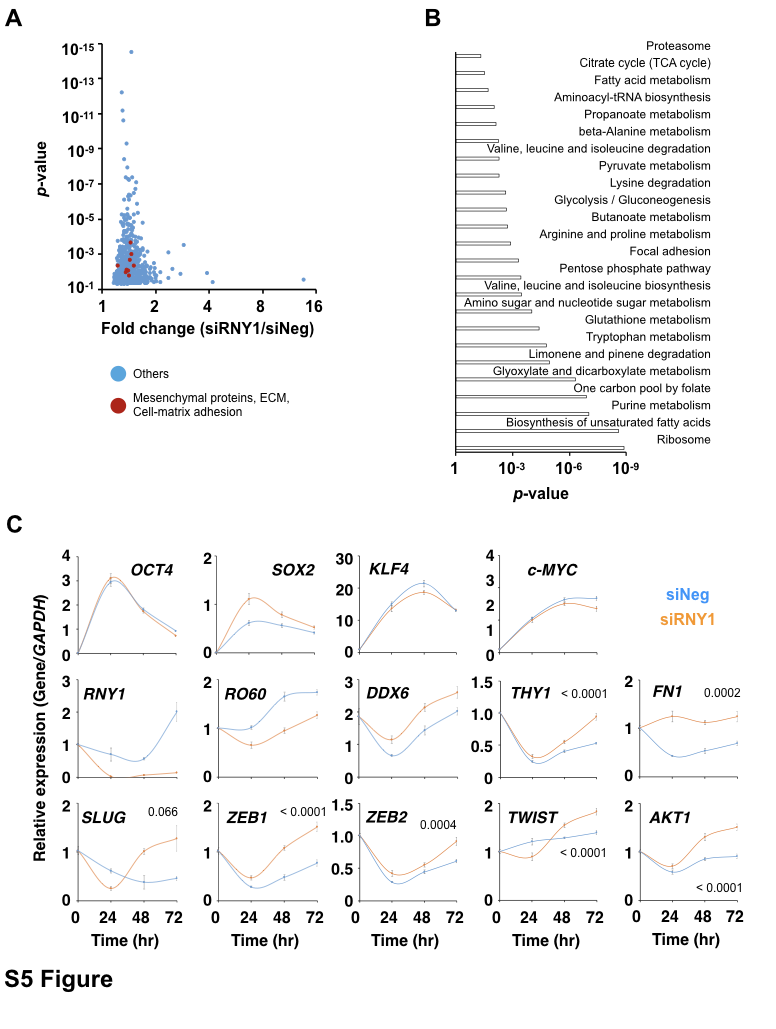

Supplement: S5 Fig — (A) Global proteomics using the iTRAQ method. Red dots indicate MET-related proteins, e.g., mesenchymal, ECM, and cell-matrix adhesion proteins. (B) Proteins were categorized based on biological processes using Gene Ontology (GO) annotations. (C) mRNA expression in OSKM- and siRNA-treated TIG-1 fibroblasts from 0 to 72 h. Individual RNA expression levels were normalized to GAPDH expression levels. Data are presented as means ± SEM. (TIFF) [file pone.0203708.s005.tiff]

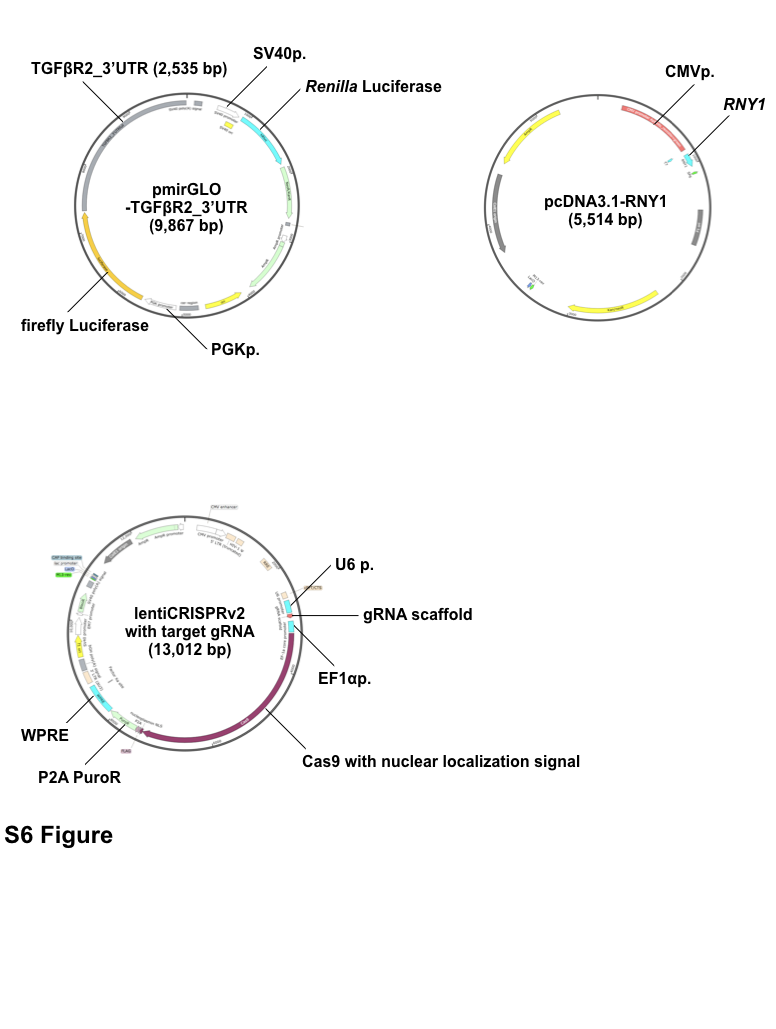

Supplement: S6 Fig — (TIFF) [file pone.0203708.s006.tiff]

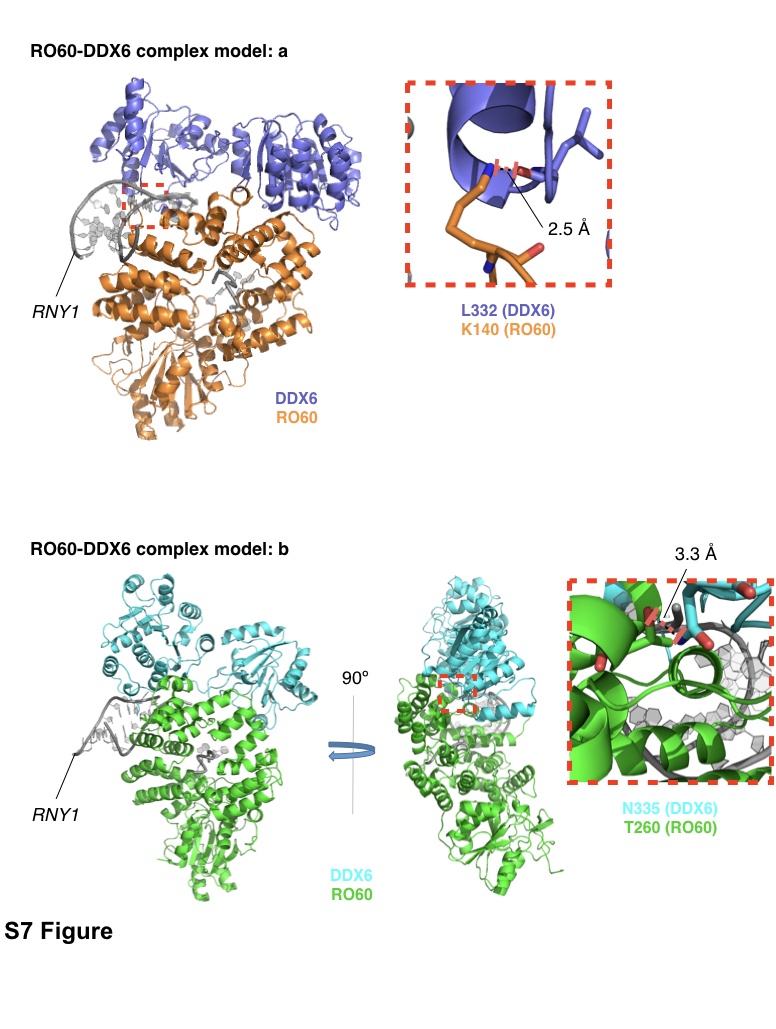

Supplement: S7 Fig — The proposed two structure models of hRO60 and DDX6 complex. The molecular structures of hRO60, RNY1 and DDX6 are colored as mocha, gray and aqua in (a), and green, gray and cyan in (b). The expected hydrogen bonds and their residues are represented as dashed lines and sticks, respectively, in close up views of the expected interaction between hRO60 and DDX6 in the presence of RNY1. (TIFF) [file pone.0203708.s007.tiff]

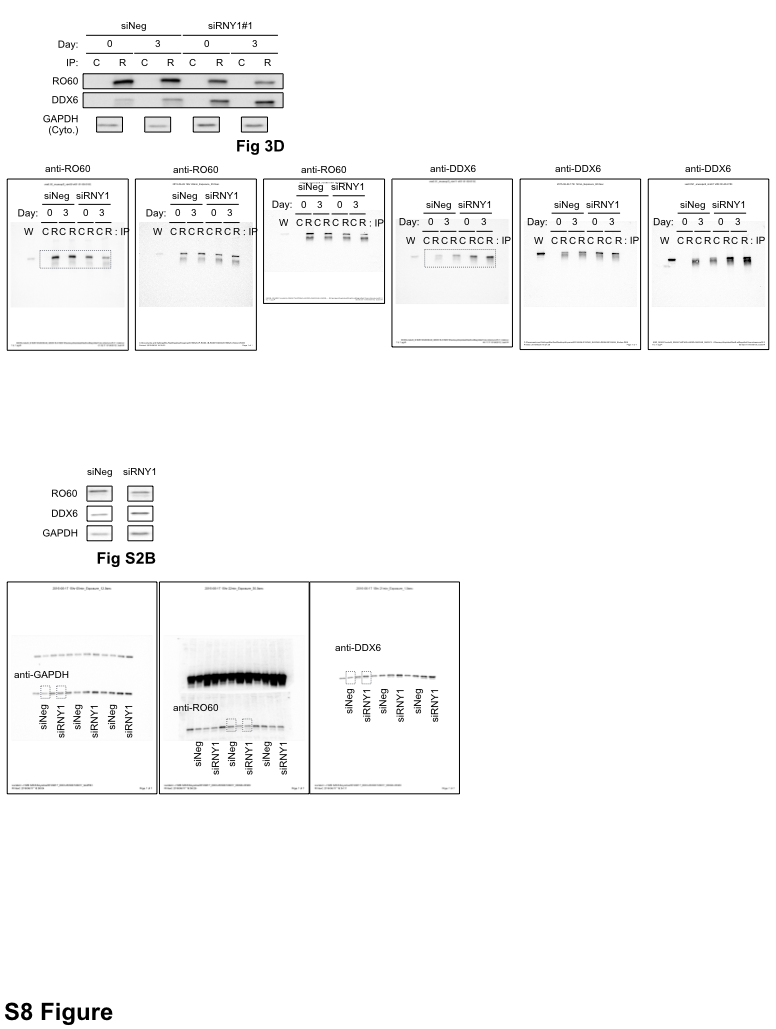

Supplement: S8 Fig — Raw blotting data in Fig 3D, and Part B of S2 Fig. The dotted-squares indicate the blotting bands used in each figure. (TIFF) [file pone.0203708.s008.tiff]
